# Targeting a genomic RNA G-quadruplex of dengue virus with small molecules as an alternative to protein-targeted therapeutics

**DOI:** 10.1186/s12929-026-01262-x

**Published:** 2026-05-27

**Authors:** Yeong Jun Kim, Moumita Das, JunYoung Song, Shreyasi Das, Han-Jun Kim, Jeong-Ki Kim, Ki-Young Lee, Kyeong Kyu Kim, Hye-Ra Lee

**Affiliations:** 1 Department of Biotechnology and Bioinformatics, College of Science and Technology, Korea University, 2511 Sejong-Ro, Sejong, 30019 Republic of Korea; 2 Department of Precision Medicine, Graduate School of Biomedical Science (GSBMS), Sungkyunkwan University School of Medicine, Suwon, 16419 Republic of Korea; 3 College of Pharmacy, Korea University, Sejong, 30019 Republic of Korea; 4 Department of Pharmacy, Sungkyunkwan University School of Medicine, Suwon, 16419 Republic of Korea; 5 Institute for Antimicrobial Resistance and Therapeutics, Sungkyunkwan University School of Medicine, Suwon, 16419 Republic of Korea; 6 Department of Lab Medicine, College of Medicine, Korea University, Seoul, 136-701 Republic of Korea

**Keywords:** Dengue virus, G-quadruplex, BRACO-19, Antivirals, Flaviviruses

## Abstract

**Background:**

Given the increasing global incidence of dengue virus (DENV) infections and the lack of approved antiviral therapies, developing a new antiviral strategy against DENV is urgently required. However, despite considerable efforts to develop protein-targeted antivirals, clinical translation has largely failed, highlighting the need for alternative, non-protein targets.

**Methods:**

Here, we investigated a novel therapeutic approach that targets RNA G-quadruplexes (G4s), highly conserved secondary structures across viral strains, including all serotypes. Bioinformatic predictions and biophysical analyses identified conserved G4-forming sequences within the DENV genome.

**Results:**

Among the tested representative G4-binding ligands, BRACO-19 showed the highest stabilizing effect on G4 structures, particularly at the G4-3 region corresponding to the NS3 gene with high binding affinity. Functionally, BRACO-19 treatment significantly reduced viral translation resulting in strong antiviral effects in a mouse model of DENV infection. Consistently, recombinant DENV carrying a G4-3-disrupting mutation showed enhanced viral gene expression and attenuated sensitivity to BRACO-19. These findings establish DENV RNA G4 as a druggable structural element and position BRACO-19 as a promising lead compound for dengue therapeutics. Furthermore, we also demonstrated that BRACO-19 exhibits broad-spectrum activity against all DENV serotypes. This RNA structure-based approach offers an alternative to protein-targeted therapeutics, helping to mitigate mutational escape and serotype variability in dengue virus.

**Conclusion:**

Collectively, our study highlights a novel therapeutic strategy that directly targets conserved RNA secondary structures, paving the way for the development of broad-spectrum antivirals against flaviviruses by targeting a non-canonical nucleic acid structure.

**Supplementary Information:**

The online version contains supplementary material available at 10.1186/s12929-026-01262-x.

## Background

Dengue virus (DENV), a positive-sense single-stranded RNA of the *Flaviviridae* family, is one of the most widespread vector-borne pathogens in the world [57]. Comprising four genetically and immunologically distinct serotypes, DENV is estimated to infect between 280 and 550 million people annually, resulting in approximately 25,000 deaths reported each year [3]. In 2024, the World Health Organization (WHO) reported approximately 6.3 million suspected DENV cases in Brazil alone, underscoring an increase in global incidence. This increase has worsened due to climate change, which has expanded the ecological range of *Aedes* mosquito vectors and contributed to sustained transmission in both endemic and emerging regions [49]. Following host entry via receptor-mediated endocytosis initiated by the interaction of the viral envelope (E) protein with host receptors such as DC-SIGN, the DENV genome is released into the cytoplasm, where it is translated into a single polyprotein [23]. This polyprotein is cleaved into three structural proteins (C, prM/M, and E) and seven nonstructural proteins (NS1-NS5) [33]. Among these, NS3 functions as a serine protease and helicase, and NS5 acts as the RNA-dependent RNA polymerase (RdRp) responsible for viral genome replication [33]. Newly assembled virions mature and are released to infect additional cells. Notably, DENV transmission is remarkably efficient, estimated to be over twice that of SARS-CoV-2, driven by both mosquito-mediated random transmission and antibody-dependent enhancement (ADE), which can increase disease severity upon secondary infection [18]. Given these transmission dynamics and limited efficacy of current serotype-specific vaccines, the development of broad-spectrum antiviral agents remains a pressing and urgent priority.

To date, most antiviral strategies against DENV have primarily targeted viral proteins, especially NS3 and NS5 [42]. However, these approaches are limited by weak inhibitor binding affinity, conformational flexibility, and the high mutation rates characteristic of RNA viruses [35, 51], which facilitate the rapid emergence of drug-resistance variants. These challenges have prompted growing interest in alternative antiviral strategies that target more conserved and structurally stable elements of the virus. One promising avenue involves guanine (G)-rich sequences within viral RNA that can fold into G-quadruplexes (G4s), thermodynamically stable, non-canonical four stranded secondary structures formed by stacks of G-tetrads (G·G·G·G) stabilized by monovalent cations [43]. G4s are structurally polymorphic and adopt diverse topologies, enabling specific interactions with metal ions, small molecules, and proteins [63]. Through these interactions, G4s regulate transcription, translation, and genome replication [60]. While G4s have been extensively studied in DNA viruses, their roles in RNA viruses are less well defined. Accordingly, G4s are considered as a drug target since structural modulation of G4 significantly affects the transcriptional/translational machinery. For example, in Zika viruses, G4 stabilization in envelope protein-coding regions reduces viral replication in vitro [39]. In another member of the *Flaviviridae* family, Tick-borne encephalitis virus (TBEV), a G4 element located at the NS4B/NS5 junction has been shown to be a critical element for TBEV replication [24]. These findings consistently support the idea that G4s in the RNA virus represent promising targets for the antiviral drug discovery. However, despite mounting evidence of G4-mediated regulation in other RNA viruses, studies in DENV have largely been limited to in vitro biophysical analyses, leaving their functional significance within the viral genome unresolved [53]. Although DENV G4 has been proposed as a potential antiviral target, its roles during infection and the mechanism underlying their activity remain undefined. Therefore, a systematic investigation of potential G4 elements within the DENV genome is required to elucidate their structural features and biological functions, which will be essential for the rational development of G4-targeted therapeutics against dengue.

In this study, we identified eleven putative G4-forming sequences in the DENV2 genome, seven of which are located on the positive strand. Biophysical analyses revealed that these RNA motifs form parallel or hybrid G4 topologies. Among several G4-stabilizing ligands tested, BRACO-19 demonstrated the highest selectivity and stabilizing activity, particularly toward the G4-3 motif located within the NS3 coding region. Functional assays further demonstrated that BRACO-19 suppresses DENV2 translation and subsequently reduces viral replication, consistent with its stabilizing effect on the G4-3 structure. A recombinant DENV2 mutant in which the G4-3 motif was disrupted showed increased gene expression and viral replication, providing additional confirmation of this structure’s functional relevance. In vivo studies using AG129 mice demonstrated the antiviral efficacy of BRACO-19 against DENV2. Moreover, BRACO-19 inhibited viral propagation by interfering with NS3 gene expression across all four DENV serotypes, indicating its potential as a broad-spectrum antiviral agent against flaviviruses. Collectively, these findings provide the first functional evidence of G4 structures in the DENV context and highlight G4 stabilization as a promising strategy for broad-spectrum antiviral development.

## Methods

### Prediction of G4-forming sequences from the DENV genome

To identify putative G4-forming sequences within the DENV genome as potential therapeutic targets, a computational prediction approach was employed. Sequences were selected based on stringent criteria: (i) no bulges or mismatches and (ii) a minimum pqsfinder score of 25, in order to enrich for high-confidence G4 candidates likely to fold under physiological conditions. The expected outcome of this analysis was a shortlisted set of conserved, high-scoring G4 loci distributed across the DENV genome, with conservation across serotypes serving as an additional filter for their biological relevance. G4 prediction was performed on the DENV2 reference genome obtained from National Center for Biotechnology Information (NCBI) (Reference Sequence: NC_001474) using the pqsfinder package in R v4.4.2 [25]. G4 with no bulges or mismatches and a minimum score of 25 were selected for further analysis. The plot of G4 frequency per unit length, generated in Python v3.11, is an overview of the putative G4 distribution throughout the DENV2 genome. To assess the conservation of the final set of G4 across other DENV serotypes, the sequences were aligned with reference genomes of the other remaining three serotypes (NCBI Reference Sequence: NC_001477, NC_001475, NC_002640) of DENV using Clustal O v1.2 [36, 54]. The aligned sequences of strains in four DENV serotypes were downloaded from G4-Virus [31], a G4 database for human viruses. Additionally, to observe the degree of conservation of these seven G4s in other flaviviruses, conservation analysis on a set of representative sequences from 67 other members of the flavivirus family was conducted. These sequences for other flaviviruses were downloaded from NCBI as well. Sequence conservation for each G4 was visualized by generating sequence logos created with Web Logo 3 application v3.7.12 [10].

### Chemicals and antibodies

Small molecule ligands included PDS (pyridostatin; 18,013, Cayman Chemical), BRACO-19 (N,N′-(9-(4-(dimethylamino)phenylamino)acridine-3,6-diyl)bis(3-(pyrrolidin-1-yl) propenamide); GC50140, GLPBIO), PhenDC3 (3,3′-[1,10-Phenanthroline-2,9-diylbis(carbonylimino)]bis[1-methylquinolinium]1,1,1-trifluoromethanesulfonate; CS-7711, Chemscene), TMPyP2 (5,10,15,20-tetra-(N-methyl-2-pyridyl)porphyrin; T40846, Frontier Scientific), Quercetin (117-39-5, MedChemExpress), TMPyP4 (HY-10443, MedChemExpress), and Thioflavin T (obtained from Collaborator). For immunoblotting (IB), the following antibodies were used: anti-DENV NS1 (ARG65660, Arigo Biolaboratories; 1:4000), anti-DENV NS3 (ARG66682, Arigo Biolaboratories; 1:3000), anti-DENV NS5 (NBP2-42,900, Novus Biologicals; 1:3000), and anti-DENV Capsid (GTX103343, GeneTex; 1:3000). The 3H5-1 and 4G2 monoclonal antibodies, purified from hybridoma supernatants by YNTOAB, were used for flow cytometry (1:100). Goat anti-Mouse IgG-Alexa Fluor 488 (109-546-006, Jackson ImmunoResearch) was used as the secondary antibody for flow cytometry (1:250).

### Oligonucleotides

The RNA oligonucleotides used for CD spectroscopy and T_m_ melting experiments were purchased from BIONEER, dissolved in nuclease-free water to stock concentration of 100 µM, and stored at − 20 °C until use (Table S2). Oligonucleotides and primers for cloning G4 fragments into plasmids, as well as for amplifying these fragments for in vitro assays, were obtained from Macrogen, reconstituted in nuclease-free water to 100 µM, and stored at − 20 °C until further use.

### Cells and viruses

Vero76 and 293FT cells were maintained in Dulbecco’s Modified Eagle’s Medium (DMEM) supplemented with 10% fetal bovine serum (FBS) and 1% penicillin–streptomycin (P/S) at 37 °C in a humidified incubator with 5% CO₂. The 3H5-1 hybridoma cells were kindly provided by the Korea Research Institute of Chemical Technology (KRICT), and the 4G2 hybridoma cells were obtained from the American Type Culture Collection (HB-112, ATCC). Hybridoma cells were cultured in RPMI 1640 medium supplemented with 10% FBS and 1% P/S. DENV serotypes 1–4 were obtained from the Korea Bank for Pathogenic Viruses (KBPV). For virus propagation, Vero76 cells were infected at a MOI of 0.01 and maintained in DMEM containing 2% FBS for 6–7 days. When approximately 70% cytopathic effect (CPE) was observed, aliquoted, and stored at − 80 °C for use in subsequent experiments. Viral titer was measured by focus-forming assay (FFA).

### Focus forming assay

This immunostaining-based assay quantifies infectious viral particles by counting discrete foci of infected cells, each representing an individual infectious unit. Viral titers are expressed as focus-forming units per milliliter (FFU/mL). Conceptually, this method is analogous to a plaque assay; however, it relies on antibody-based detection of infected cells rather than cell lysis, enabling more sensitive and rapid quantification of infectious viruses [37]. Briefly, Vero76 cells were seeded in 96-well plates and infected with tenfold serial dilutions of the virus. After 24 h of incubation, the cells were fixed with 4% paraformaldehyde and permeabilized using 0.1% Triton X-100. Immunostaining was performed using an anti-DENV E protein antibody [4G2], followed by FITC-conjugated secondary antibody. Foci were visualized and counted, and the viral titer (FFU/mL) was calculated with following formula: (Number of foci X dilution factor) / volume of inoculum.

### Time-of-addition assay

The time-of-addition assay is used to infer which stage of the viral life cycle is preferentially targeted by a compound by varying the timing of drug exposure relative to infection and assessing the resulting antiviral effect. Depending on the timing of drug addition, this approach allows discrimination between effects on early events, including viral entry, uncoating, and translation, and later stages, including RNA replication, assembly, and release. To determine the specific stage of the DENV life cycle targeted by BRACO-19, a time-of-addition assay was performed based on a previously described method [11]. Briefly, Vero76 cells were infected with DENV at MOI 1.0. BRACO-19 was added to the culture medium at the indicated time points (− 2, 0, 2, 4, 8, 12, and 24 h) relative to infection. Cells were harvested at 24 h post-infection (hpi), and the percentage of DENV-infected cells was quantified by flow cytometry.

### Cycloheximide chase assay

To dissect whether BRACO-19 primarily affects de novo viral protein synthesis or downstream steps of the DENV2 life cycle, a cycloheximide (CHX) chase assay was performed. Vero76 cells were seeded in 6-well plates and infected with DENV2 (MOI 1.0) for 1 h, followed by washing and replacement with fresh complete medium. For the early-treatment condition, BRACO-19 (25 µM) or CHX (10 µg/mL) was added immediately after the infection period. For the late-treatment condition, infected cells were first incubated for 24 h to allow accumulation of nonstructural proteins, after which BRACO-19 (25 µM) or CHX (10 µg/mL) was added. At the indicated time points after drug addition (0, 4, 8, and 16 h), cells were harvested for parallel analysis of viral RNA and protein. Total RNA was extracted, and DENV2 genomic RNA copy numbers were quantified by RT-qPCR targeting the conserved 3′ UTR. In parallel, cell lysates were prepared in NP-40 lysis buffer, and equal amounts of protein were subjected to SDS-PAGE and immunoblotting for NS5 and NS1.

### Cytotoxicity assay

Vero76 cell was seeded on 96-well plate and treated with BRACO-19 at concentrations ranging from 3.1 to 400 µM twofold serially diluted in DMEM serum-free media for 48 h. After treatment, media was changed with MTT in phenol Red/serum-free DMEM (3 mg/mL) for 4 h. Following this, media was aspirated and DMSO was added. Absorbance was measured at 540 nm using a microplate reader (Varioskan Lux, Thermo Scientific).

### Immunoblotting (IB)

Vero76 cell was seeded in 24-well plate and infected with DENV for 24 h and lysed in 0.1% NP-40 buffer. Equal protein amounts were resolved by SDS-PAGE, transferred to PVDF membranes, and probed with primary antibodies against NS1, NS3, NS5, and Capsid. Membranes were then incubated with HRP-conjugated secondary antibodies, and proteins were detected by chemiluminescence. Band intensities were quantified using ImageJ.

### Flow cytometry

Vero76 cells were seeded on 24-well plate and infected with DENV for 24 h. Infected cells were harvested, washed with cold PBS, fixed in 70% ethanol, and permeabilized with 0.1% Triton X-100. Cells were stained with DENV E-targeting primary antibodies (3H5-1 or 4G2) followed by Alexa Fluor 488-conjugated goat anti-mouse IgG. After PBS washes, samples were analyzed using CytoFlex flow cytometer (Beckman Coulter).

### RNA isolation and RT-PCR

DENV-infected cells were lysed in TRIzol, and total RNA was extracted by chloroform/isopropanol precipitation, washed with 70% ethanol, and dissolved in DEPC-treated water. cDNA was synthesized using ReverTra Ace qPCR RT Master Mix (Toyobo) according to the manufacturer’s instructions. Quantification of viral genome copies was performed by RT-PCR targeting a conserved region within the DENV 3′ UTR region, as previously described [7], using the following primer pair: Forward, 5′-GGTTAGAGGAGACCCCTCCC-3′; Reverse, 5′-GGCGTTCTGTGCCTGGA-3′.

### Saturation transfer difference NMR spectroscopy and chemical shift perturbation (CSP) analysis

To map the specific binding epitope of BRACO-19 on G4-3 RNA and confirm direct intermolecular contact at atomic resolution, STD-NMR and CSP analyses were conducted. STD-NMR was selected because it is well-suited for detecting the binding of small molecules to large biomolecular targets like proteins and nucleic acid structures, and can identify which protons of the ligand are in closest proximity to the RNA surface [55]. CSP analysis was included to complement STD-NMR by tracking chemical shift changes in BRACO-19 proton resonances upon RNA addition, allowing residue-specific identification of the binding interface. All NMR samples were prepared in a buffer containing 10 mM Tris, pH 7.5, 100 mM KCl, and 10% D2O. BRACO-19 and RNA were mixed at final concentrations of 0.5 mM and 0.025 mM, respectively, corresponding to a 20:1 molar ratio of ligand to RNA. The concentration ratio was decided after optimizing different ratios. Higher concentration of drug caused aggregation formation. All NMR experiments were performed on a 850 MHz Cryo NMR spectrometer at the National Center for Inter-University Research Facilities (NCIRF), Seoul National University. For STD-NMR experiments, spectra were acquired for BRACO-19 in the absence and presence of RNA. Selective saturation was applied at on-resonance frequencies of 4.9, 5.0, 5.5, 5.9, and 6.5 ppm, while irradiation at an off-resonance frequency of 40 ppm was used as a reference. Among these conditions, irradiation at 4.9 ppm was found to provide the clearest difference between the two spectra and was therefore selected for further analysis. A train of selective saturation pulses was applied for a total saturation time of 2.0 s. The reduction in signal intensity observed under on-resonance irradiation was interpreted as evidence of intermolecular contact between BRACO-19 and RNA. For chemical shift perturbation (CSP) analysis, 1D 1H NMR spectra of BRACO-19 were recorded in the absence and presence of RNA using the same final concentrations of BRACO-19 (0.5 mM) and RNA (0.025 mM). The proton resonances of BRACO-19 were assigned on the basis of two previously published studies [55, 58] and with the aid of an NMR chemical shift prediction program [6].

### Production of DENV2-GFP recombinant virus

The full-length genome of DENV2-GFP was divided into seven fragments (F1-F7), PCR-amplified, and cloned into the pUC18 vector. An additional fragment containing a CMV promoter and a poly(A) signal was included. All fragments were PCR-amplified and purified prior to assembly. Full-length viral genomes were reconstructed using a circular polymerase extension reaction (CPER)–based strategy, as previously described, in which overlapping DNA fragments are assembled in a single in vitro reaction. Each fragment contains terminal overlaps that enable seamless assembly into a closed viral genome. This approach was used to generate recombinant DENV2-GFP viruses harboring either the wild-type or G4-disrupting mutant version of the G4-3 sequence. Briefly, 0.1 pmol of each purified fragment was used as template DNA. CPER under the following conditions: 98 °C for 10 s and 68 °C for 14 min for 35 cycles. CPER products were transfected into 293FT cells and co-cultured with Vero76 cells at 12 h post-transfection. After 14 days, when approximately 70% CPE was observed, supernatants were collected, aliquoted, stored at − 80 °C, and viral titers were determined by focus-forming assay.

### MD simulation study

To evaluate the structural dynamics of DENV G4-3 and the molecular basis of ligand-mediated G4 stabilization, all-atom molecular dynamics (MD) simulations were performed. This approach was chosen because MD captures conformational flexibility, G-quartet planarity, and ligand-binding modes at atomic resolution. Systems were designed to directly compare wildtype (G4-3WT) versus mutant, (G4-3MT) to establish structure dependence and to contrast the binding of BRACO-19 with two alternative binders, PhenDC3 and Quercetin under identical conditions. RMSD and RMSF metrics serve as quantitative readouts of structural stability and conformational dynamics, reflecting G4 integrity, system equilibration, and ligand-induced structural fluctuations. Lower RMSD and more favorable binding free energies in the WT relative to the MT model are expected to reflect G4-structure-dependent ligand interaction. The initial mixed-topology DNA G4 structure (G4-3) was generated using the 3D-NUS server [44], consistent with the circular dichroism (CD) spectra observed for hybrid G4s. RNA G4 was obtained by converting the deoxyribose to ribose using PyMOL v2.5 (Schrödinger, L. and DeLano, W. (2020) PyMOL). A K + ion was manually placed at the G-quartet core to stabilize the structure. BRACO-19 (CID 9808666) was retrieved from PubChem [29], converted from SDF to PDB with Avogadro v1.2 [20], and parameterized using GAFF via antechamber [64]. RNA was parameterized with the bsc1 force field with vdW correction [65]. Four systems of G4-3WT and G4-3MT (with and without BRACO-19) and additional G4-3 + PhenDC3 and G4-3 + Quercetin systems were prepared in GROMACS v2023.2 [62] under periodic boundary conditions. Complexes were solvated in a cubic box with a fixed distance of 1 nm between the solute and the box using TIP4P-Ew water [26] and 100 mM KCl was added to approximate the ionic strength in the system; in ligand-containing systems, ligands were randomly inserted into the solvent box prior to solvation. Energy minimization was performed using steepest descent for up to 50,000 steps. Long-range electrostatic interactions were calculated by Particle Mesh Ewald (PME) [13] with 0.16 nm grid spacing. A 1.2 nm cutoff was applied for short-range van der Waals, SHAKE algorithm was used to stabilize the motion of hydrogens [50] and LINCS [22] was used for bond constraints. Equilibration was run under NVT and NPT ensembles with positional restraints (1.0 kcal mol-1 Å-1) for 100 ps each, followed by 500 ns production MD at 300 K and 1 atm with 2 fs integration timestep. Binding free energy was estimated from the last 300 ns using gmxMMPBSA [61], RMSD of G4 tetrads was calculated relative to the starting structure, RMSF of G4 residues and G4-ligand residue-specific interaction heatmap were generated to compare the persistent residue contacts between BRACO-19, PhenDC3 and Quercetin. The final structures were visualized in PyMOL. The 3D interaction figures for ligand binding to G4-3 were generated in PyMOL.

### Circular dichroism spectra and T_m_ melting analysis

Circular Dichroism (CD) spectroscopy and Thermal melting T_m_ analysis were performed to characterize the G4 topology and thermodynamic stability of seven candidate DENV G4 forming sequences, and to evaluate the effect of G4-targeted ligands on their structural conformation and stability, a prerequisite for interpreting further downstream functional assays [52]. CD spectroscopy was selected as the primary biophysical tool because the G4 folds show characteristic topological peaks, in which a dominant positive band near 260 nm with a negative peak near 240 nm is indicative of a parallel G4, a positive band near 295 nm with a negative band near 260 nm indicates an antiparallel topology, and mixed signatures indicate a hybrid/mixed form [40]. Thermal melting monitored at the characteristic 263 nm peak establishes the structural stability of each G4 and quantifies ligand induced stabilization imparted by each ligand [ΔT_m_ = T_m_ (with ligand) − T_m_ (without ligand)]. CD spectral analysis and T_m_ melting analysis were performed on a J-810 spectropolarimeter (JASCO) equipped with a CDC-426F Peltier function temperature controller. RNA G4 (15 μM) was prepared in 100 mM KCl and 10 mM Tris–HCl buffer by heating at 95 °C for 10 min, followed by gradual cooling to 4 °C at 0.5 °C/min. For ligand-binding studies, RNA G4 was incubated with drugs at a 1:2 molar ratio (15 μM: 30 μM) for 30 min prior to measurements. CD spectra were recorded from 220 to 320 nm using a 1 mm quartz cuvette with 200 μL sample volume, scan speed of 100 nm/min, data pitch of 1 nm, and bandwidth of 0.2 nm. Thermal melting was monitored at 263 nm from 20 to 95 ℃ with a heating rate of 1 ℃/min and data pitch of 0.5 ℃. Data were analyzed and plotted using GraphPad Prism 8.3.0.

### ^1^H Nuclear magnetic resonance spectroscopy

One-dimensional (1D) 1H NMR spectroscopy was performed to provide direct solution-state evidence for G4 formation in G4-3, confirming the CD data with atomic resolution information. The criterion for G4 detection by NMR is the appearance of Hoogsteen hydrogen-bonded imino proton signals in the region of 10–12 ppm [39]. NMR experiment was performed using 700 MHz BRUKER NMR spectrometer (Bruker, Germany). The sample was prepared using methodology as mentioned in the previous section of CD spectral analysis. The 1D ^1^H NMR spectra were recorded using 450 μM G4-3 in 10% D_2_O solvent at room temperature (25 ℃). NMR data were processed and analyzed by SpinWorks v4.0. software.

### Fluorescence titration assay

To quantify the binding affinity of the G4 ligand BRACO-19 for G4-3WT and G4-3MT, fluorescence titration assays were performed based on the intrinsic fluorescence enhancement of BRACO-19 upon G4 binding- a well-established approach for measuring G4-ligand dissociation constants (*K*_D_) without the need for intrinsic labelling [2]. Comparing affinities between G4-3WT and G4-3MT allowed direct assessment of the G4-structure dependent binding. The assay was performed in 96-well plate using a Synergy H1 multi-well plate reader. BRACO-19 (10 μM) was added to twelve wells, and RNA G4 was titrated by serial dilution (0–25 μM), while the twelfth well served as a blank. Fluorescent emission enhancement was recorded at 440 nm (λ_ex_ = 360 nm). Data were normalized to the blank (∆F = F_Sample_ − F_Blank_) and fluorescence enhancement curves were generated by fitting ∆F against RNA G4 concentration using a two site (total and non-specific) non-linear regression model in GraphPad Prism.

### Construction of reporter system for in vitro coupled transcription/translation (IVT) Assay

The reporter construct was generated using the pEYFP-N1 plasmid. This IVT reporter system was used to assess the effect of DENV G4 motifs stabilization on translation efficiency of a downstream reporter. In this system, reduced YFP expression in the presence of a G4 ligand reflects translational repression independent of transcription. A T7 promoter was inserted to enable compatibility with the TnT® Quick Coupled Transcription/Translation Systems (Promega). G4 fragments were cloned downstream of the ATG start codon in the sense strand. Point mutations in G4 fragments were introduced by Phusion PCR using the WT construct as a template. The IVT assays were performed according to the manufacturer’s instructions.

### In vitro coupled transcription/translation (IVT) Assay

To directly assess the functional consequences of G4 stabilization on viral RNA translation, a critical step in DENV replication, a cell-free IVT reporter system based on the pEYFP-N1 plasmid was used. This IVT reporter system was designed to specifically monitor how the stabilization of individual DENV G4 motifs affects the translation efficiency of a downstream reporter protein, such that reduced YFP expression in the presence of a G4 ligand is interpreted as G4-dependent translational repression. A T7 promoter was inserted to enable compatibility with the TnT® Quick Coupled Transcription/Translation System (Promega). Individual G4 fragments were cloned downstream of the ATG start codon in the sense strand [15, 38] using Gibson assembly, ensuring direct G4-ribosome encounter during elongation. To confirm that the observed translational effects were G4 structure-based and not sequence-based, point mutations were introduced into G4 fragments by Phusion PCR, using the G4-3WT construct as a template to create G4-3MT. The IVT assay was performed according to the manufacturer’s protocol, and the resulting protein was quantified using western blot analyses. The reduced YFP signal corresponding to G4-3WT in the presence of BRACO-19 is interpreted as evidence of G4-mediated translational repression, while the equivalent signal across constructs in the absence of ligand or in the mutant confirms that sequence context alone does not impair translation.

### In vitro transcription assay

To verify that any reduction in YFP output observed in the IVT reporter assay reflects translational repression and not transcriptional repression, a standalone in vitro transcription assay was performed to assess whether the presence of the G4-forming sequence or the addition of the BRACO-19 ligand altered the length of the mRNA transcript produced from the reporter construct. This control was necessary to demonstrate that full-length mRNA was produced, attributing translational differences specifically to G4-mediated ribosome stalling. The reporter construct was transcribed using the T7 RiboMAX™ Express Large Scale RNA Production System (Promega), and the length of the produced mRNA was analyzed using agarose gel electrophoresis. The single, full-length mRNA band in the absence and presence of BRACO-19 is interpreted as evidence that neither G4 sequence context nor the ligand interferes with transcription, proving the IVT assay as a reliable readout of translational regulation.

### BRACO-19 in vivo experiment

To evaluated the in vivo efficacy of BRACO-19 against DENV2 infection, six- to seven-week-old female AG129 mice were intraperitoneally inoculated with 1 × 10^6^ FFU of DENV2. BRACO-19 was administered by intraperitoneal injection at 5 mg/kg daily. Body weight was monitored, and orbital blood was collected at designated time points for quantification of viral RNA copy numbers using 3′ UTR primers. 15 days post-infection, mice were euthanized for histopathological examination of major organs.

## Results

### Identification and validation of G4s in the DENV2 genome

**Fig. 1:**
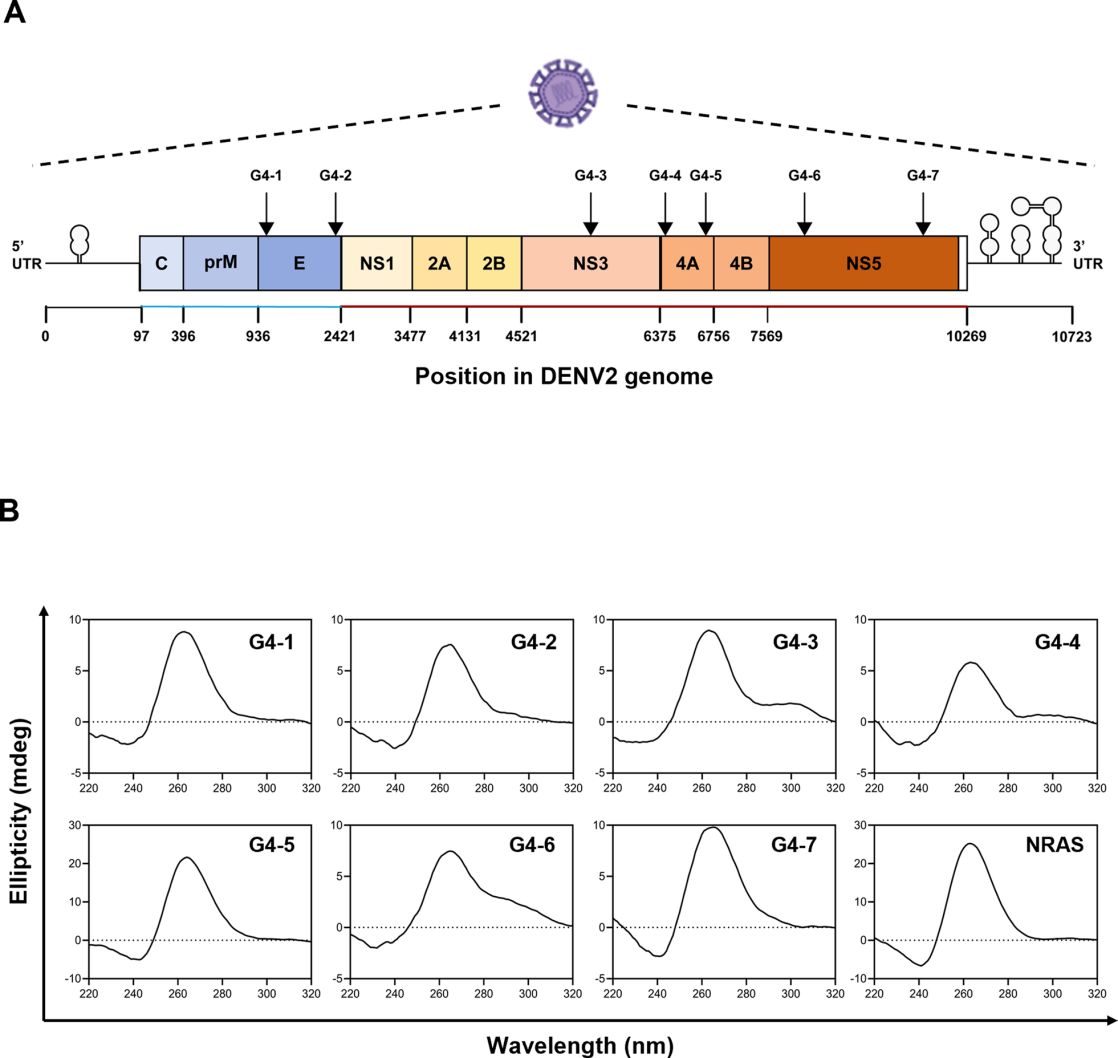
The DENV2 genome contains RNA G4 structures with both parallel and mixed topologies. **a** The diagram represents the position of protein-coding genes and seven G4 motifs used for the study (Table S2) in DENV serotype 2. The rectangular boxes indicate the approximate locations of the viral proteins, and the positions of G4s in the genome are shown with the downward arrows. **b** Comparison of CD spectra of seven G4s and NRAS for the positive control in physiological conditions. 15 µM of RNA oligonucleotides were pre-formed into G4s using 10 mM Tris–HCl (pH 7.5) and 100 mM KCl buffer. Each spectrum was an average of three accumulations in the wavelength range of 220–320 nm. The baseline correction was done with the spectra of the buffer only. The graphs were plotted and smoothed using the 12-point Savitzky-Golay algorithm. A Single peak is observed in G4-1, G4-2, G4-5, G4-7, and NRAS, whereas double peaks are in G4-3, G4-4, and G4-6

To explore the presence of RNA G4s in the DENV genome, we scanned the DENV2 reference genome (NCBI Reference: NC_001474) using the pqsfinder algorithm [25]. Among the eleven predicted putative G4-forming sequences, seven and four G4s were located on the positive and negative strands, respectively (Table S1). Given that DENV is a positive-sense single-stranded RNA virus whose genome is directly translated by host ribosomes, we focused on the seven G4s located on the positive strand. These elements were designated G4-1 through G4-7 in the 5′ to 3′ direction (Fig. 1a, Fig. S1A, and Table S2). Initial sequence alignment across representative DENV serotypes indicated that four of the seven positive-strand G4 regions (G4-2, G4-3, G4-5, and G4-6) are relatively well conserved (Fig. S1B). To further investigate the evolutionary conservation of these G4 motifs, we compared the identified regions with corresponding genomic regions across a panel of 67 flaviviruses (Table S3). In this broader analysis, only a subset of G4 motifs, most notably G4-6, showed clear conservation across multiple flaviviruses (Fig. S2A), consistent with previous reports suggesting evolutionary conservation of specific viral RNA G-quadruplex elements [59]. We further examined a subset of eight representative pathogenic flaviviruses (Table S4), including Japanese encephalitis virus (JEV), West Nile virus (WNV), Zika virus (ZIKV), Yellow fever virus (YFV), and Tick-borne encephalitis virus (TBEV) with four serotypes of Dengue virus. In this analysis, certain G4 motifs, particularly G4-2 and G4-6, exhibited partial conservation, mainly within the G-tract regions (Fig. S2B), whereas variation was primarily observed in loop regions. Importantly, conservation of the G-tract motifs has been shown to be sufficient to maintain G-quadruplex formation despite sequence variability in loop regions [15]. Therefore, even partial sequence conservation may still reflect preservation of G4 structural elements. To more comprehensively evaluate G4 conservation within DENV, we expanded our analysis to include 2,651 DENV sequences encompassing all four serotypes. This large-scale analysis yielded conservation patterns highly consistent with those observed in representative reference sequences (Fig. S1B and Fig. S3). Notably, G4-6 remained the most consistently conserved motif across the dataset, while G4-2, G4-3, and G4-5 also showed substantial conservation within DENV, particularly at the G-tract regions. Taken together, these results indicate that G4-6 is the most broadly conserved G4 motif across flaviviruses, supporting its potential as a target for pan-flaviviral therapeutic strategies. In contrast, within DENV, multiple G4 motifs (notably G4-2, G4-3, and G4-6) are well conserved and may collectively serve as suitable targets for DENV-specific intervention.

We next experimentally investigated their G4 formation and topology by conducting circular dichroism (CD) spectroscopy using synthetic RNA oligonucleotides corresponding to each of the seven G4 sequences. CD spectra revealed that G4-1, G4-2, G4-5, and G4-7 have the parallel G4 topology, displaying a strong positive peak at ~ 260 nm and a negative peak at ~ 240 nm (Fig. 1b). In contrast, G4-3, G4-4, and G4-6 contain a hybrid topology displaying dual positive peaks at ~ 260 nm and ~ 290 nm along with a negative peak at ~ 240 nm. As a control, we employed the G4-forming sequence present in the 5’ untranslated region of the *NRAS* proto-oncogene and confirmed that NRAS G4 also forms the parallel G4 topology [21]. Together, we verified that all seven predicted G4-forming sequences of DENV2 genome indeed fold into G4 structures, adopting either parallel or hybrid topologies.

### BRACO-19 suppresses DENV2 replication via G-quadruplex stabilization

G4 binding ligands have been reported to inhibit viral replication in several viruses, with their antiviral activity often correlating with G4 stabilization [1, 24, 28, 34, 39, 46]. To evaluate ligand-dependent G4 stabilization, we selected a panel of seven structurally diverse and well-characterized G4-binding ligands, including BRACO-19, Thioflavin T, TMPyP2, TMPyP4, Quercetin, PDS, and PhenDC3. These compounds represent major classes of G4-binding molecules with distinct interaction modes, such as planar stacking ligands (BRACO-19 and PhenDC3, porphyrin-based cationic binders (TMPyP2 and TMPyP4), hydrogen-bond-capable small molecules (PDS), and naturally occurring polyphenols (Quercetin). This diversity allowed us to systematically assess how different physicochemical properties influence G4 recognition and stabilization [47, 48]. At a twofold molar excess, ligand treatment altered spectral intensity and caused modest shifts in the characteristic CD peak, without substantially perturbing the overall G4 topology (Fig. S4). Notably, the extent of intensity changes varied among the seven G4s depending on the ligand, consistent with ligand-specific structural recognition and distinct binding modes [17, 52]. Among the seven ligands tested, BRACO-19 showed the most potent stabilizing effect across six of the seven G4s, with particularly pronounced ∆T_m_ increases for G4-3 (24.77 °C) and G4-6 (25.31 °C) (Table 1, Fig. S5).

**Table 1:** Melting temperature (T_m_) of DENV2 G4 oligonucleotides in the presence of different G4 ligands (RNA: ligand = 1:2)

	Melting temperature (℃, ΔT_m_)							
	NT	BRACO-19	PhenDC3	Quercetin	PDS	TMPyP2	TMPyP4	Thioflavin T
G4-1	58.54	70.91 (12.37)	64.24 (5.70)	N.D	55.25 (3.29)	55.52 (3.02)	61.71 (3.17)	56.82 (1.72)
G4-2	60.99	72.71 (11.72)	53.08 (7.91)	58.18 (2.81)	49.17 (11.82)	58.94 (2.05)	62.33 (1.34)	60.21 (0.78)
G4-3	59.31	84.08 (24.77)	N.D	43.80 (15.51)	N.D	50.52 (8.79)	N.D	60.47 (1.16)
G4-4	55.23	76.72 (21.49)	N.D	57.76 (2.53)	55.31 (0.08)	58.51 (3.28)	76.31 (21.08)	56.15 (0.92)
G4-5	66.74	N.D	N.D	N.D	66.21 (0.53)	72.37 (5.63)	N.D	66.19 (0.55)
G4-6	54.14	79.45 (25.31)	53.08 (1.06)	55.18 (1.04)	58.00 (3.86)	53.49 (0.65)	59.49 (5.35)	54.74 (0.60)
G4-7	52.44	64.68 (12.24)	61.91 (9.47)	73.50 (21.06)	55.91 (3.47)	50.69 (1.75)	54.32 (1.88)	52.02 (0.42)

**Fig. 2:**
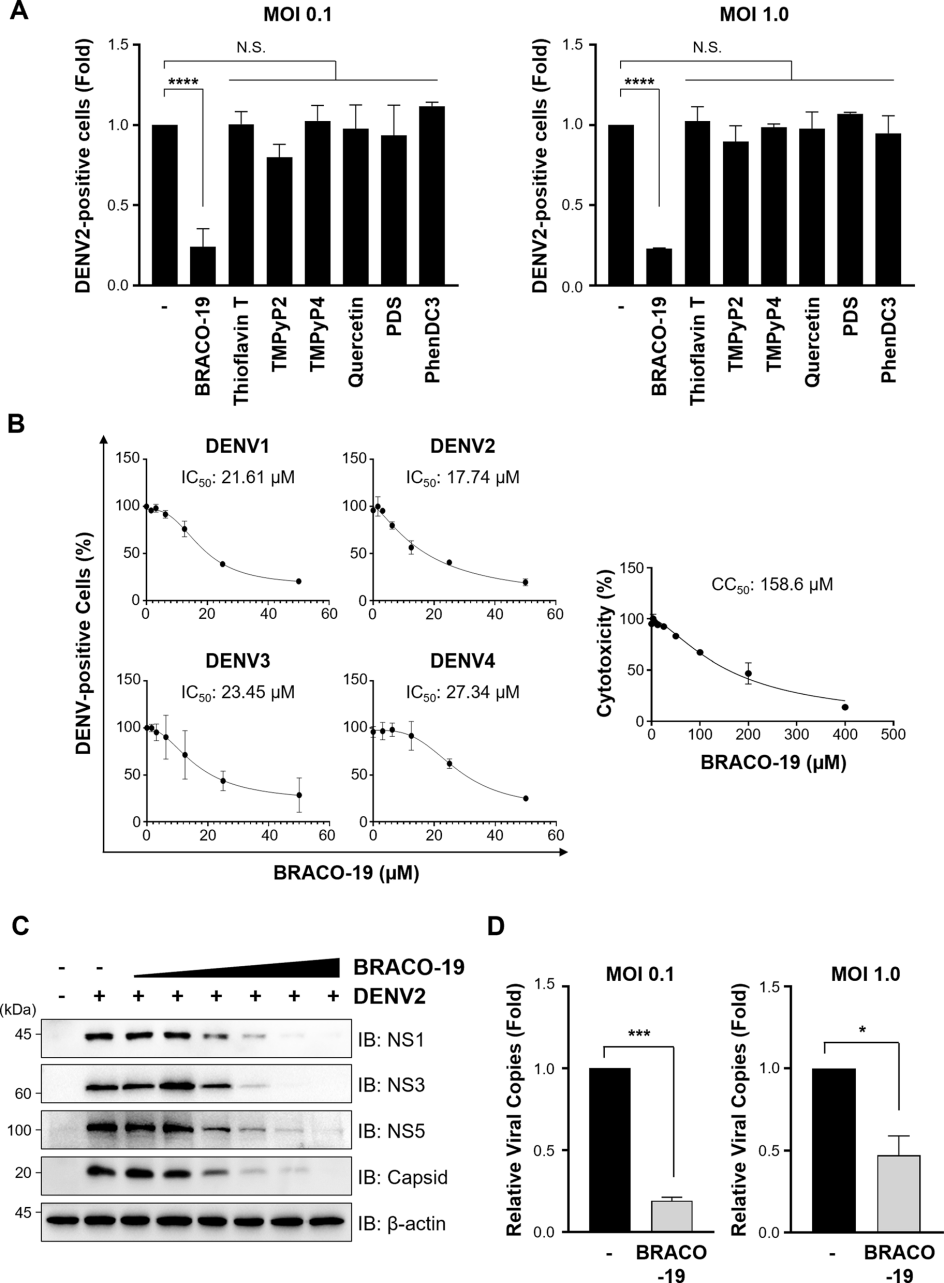
BRACO-19 shows efficient antiviral effect against DENV2. **a** Screening antiviral effect of various G4 ligands against DENV2. MOI 0.1 (Left) and 1.0 (Right) of DENV2-infected Vero76 was treated with each G4 ligand and antiviral efficacy was measured by flow cytometry. DENV was titrated by focus forming assay to assure inoculation of same amount of virus (Table S6).** b** Antiviral effect against DENV serotypes (Left) and cytotoxicity of BRACO-19 (Right). For the antiviral assay, MOI 1.0 of each serotype of DENV-infected Vero76 was treated with BRACO-19 dose-dependently and DENV-positive cells were detected by flow cytometry. For the cytotoxicity assay, Vero76 cell was treated with BRACO-19 dose-dependently for 48 h and cell survival rate was measured by MTT assay. **c** Inhibition of viral protein expression by BRACO-19. BRACO-19 was treated 1.56 µM to 50 µM twofold serially in MOI 1.0 of DENV2-infected Vero76 and NS1, NS3, NS5 and Capsid was detected by immunoblotting (IB). **d** Inhibition of viral RNA copy number by BRACO-19. MOI 0.1 (Left) and 1.0 (Right) of DENV2-infected Vero76 was treated with 25 µM of BRACO-19 and RNA samples were extracted. The RNA samples were analyzed by RT-PCR using primer targeting DENV 3’ UTR. P-value was calculated by One-way ANOVA and multiple comparison (N.S., *p* > 0.05; *p < 0.05; ***p < 0.001; ****p < 0.0001)

We next evaluated antiviral activity of seven G4 ligands in DENV-infected cells. Vero76 cells were infected with DENV2 at both high and low multiplicities of infection (MOI) and subsequently treated with individual G4 ligands. Infection levels were assessed by flow cytometry analysis using an anti-E antibody. Among tested G4 ligands, BRACO-19 exhibited the most potent antiviral effect, significantly reducing the proportion of DENV2-infected cells across MOIs (Fig. 2a). The strong antiviral effect of BRACO-19 correlated well with its highest stabilizing activity on DENV2 G4s (Fig. S4 and S5). To quantitatively assess the antiviral efficacy and cytotoxicity of BRACO-19, we conducted dose–response assays. BRACO-19 treatment yielded a half-maximal inhibitory concentration (IC_50_) ranging from 17.74 to 27.34 µM across all four DENV serotypes, and a half-maximal cytotoxic concentration (CC_50_) of 158.6 μM in Vero76 cells, indicating a favorable therapeutic window (Fig. 2b). Notably, BRACO-19 exhibited anti-viral activity against all four serotypes, with comparable IC_50_ values, suggesting broad-spectrum efficacy against DENVs (Fig. 2b). Consistent with these findings, BRACO-19 treatment resulted in a dose-dependent reduction in viral gene expression and effectively suppressed viral copy numbers (Fig. 2c, d). Taken together, our data establishes that small-molecule stabilization of conserved genomic RNA G-quadruplexes can elicit antiviral activity against dengue. In this context, BRACO-19 serves as a representative chemical probe with broad-spectrum efficacy across all four serotypes.

### BRACO-19 suppresses DENV2 replication by inducing G4-dependent translational repression

**Fig. 3:**
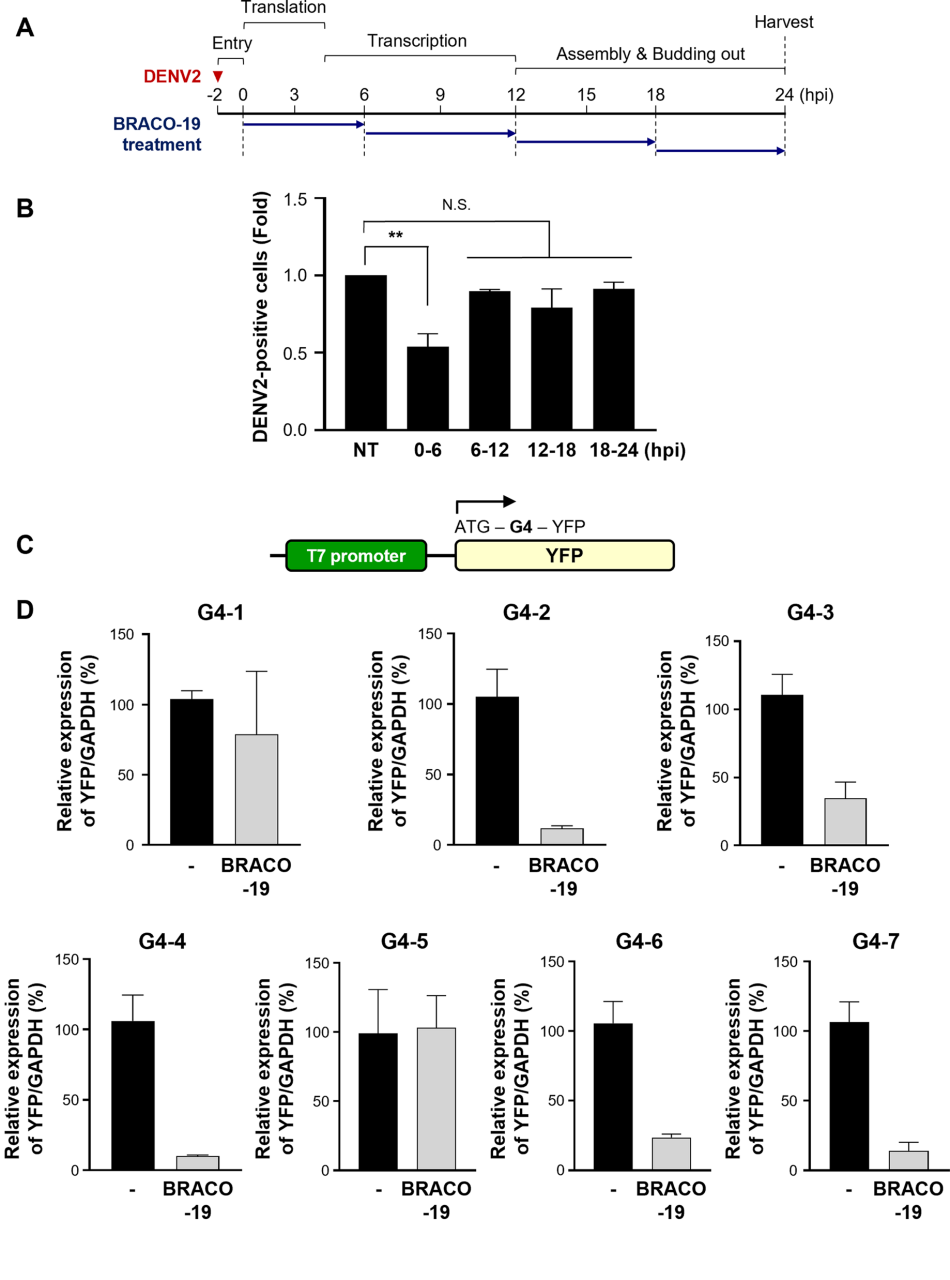
BRACO‑19 primarily targets translation during the DENV2 life cycle. **a** Schematic timeline of time-of-addition assay to identify the targeting window in DENV life cycle. The red arrowhead indicates DENV2 infection and the blue arrows indicate time points of BRACO-19 treatment. **b** Vero76 was treated with 25 µM of BRACO-19 at the indicated timepoint and MOI 1.0 of DENV2 was infected. Cells were harvested and DENV2-positive cells were measured by flow cytometry. P-value was calculated by One-way ANOVA and multiple comparison (N.S., *p* > 0.05; **p < 0.01). **c** Schematic representation of a reporter plasmid construct with G4. **d** Immunoblot analysis of YFP using IVT assay in seven G4 sequences in the presence and absence of 10 µM BRACO-19

To determine the stage of the DENV2 life cycle affected by BRACO-19, we conducted a time-of-addition experiment in which the compound was applied at various time points after infection. The antiviral effect was most pronounced when BRACO-19 was treated shortly after infection, suggesting that it interferes with early stages of the viral life cycle, such as translation and early transcription (Fig. 3a, b). To further narrow down the affected step within this early phase, we compared the effects of BRACO-19 with cycloheximide (CHX), a well-known global translation inhibitor, using chase-type experiments. When both compounds were added at 1 hpi after DENV infection, a time point at which viral translation remains active, BRACO-19 suppressed NS1 and NS5 expression to levels comparable to CHX unlike no treatment samples. Because DENV harbors positive-sense single strand RNA genome, CHX treatment, similar to BRACO-19, also reduced the copy number of DENV2 genome (Fig. S6A). In contrast, at 24 hpi, CHX markedly reduced the accumulation of NS1 and NS5, whereas BRACO-19 had minimal impact similar with time-of-addition experiment, even though the viral RNA copy numbers in both treatments were comparable to those of the untreated samples (Fig. S6B). Since CHX inhibits global translation by blocking the elongation step of protein synthesis, these results indicate that both CHX and BRACO-19 can reduce viral protein expression when applied at early time point. However, dramatically reduced antiviral efficacy of BRACO-19 at later stages suggests that its primary mechanism of action is confined to early life cycle events, particularly viral de novo translation.

Given that translation of the viral polyprotein from genomic RNA is the initial step following viral entry [23], we next investigated whether BRACO-19 primarily exerts its antiviral effect at the level of translation. For this purpose, a reporter system consisting of the pEYFP-N1 plasmid with RNA G4s following the first ATG start codon was designed. We constructed seven reporter systems, containing the seven G4s of interest, G4-1 through G4-7, in the reporter plasmid (Fig. 3c). In this system, stabilization of G4 structure by a G4-ligand is expected to interfere with the transcription and/or translation machinery, resulting in the altered YFP expression [38]. Using a coupled in vitro transcription/translation (IVT) assay followed by immunoblot analysis, YFP expression was significantly reduced in constructs containing G4-2, G4-3, G4-4, G4-6, and G4-7 (Fig. 3d). However, there was no significant change in their transcription (Fig. S8), indicating that the inhibitory effect occurred at the level of translation rather than transcription. Together with the IVT reporter assay and time-of-addition data, and consistent with CHX-like translation inhibition, these findings support a predominant role of G4-3 stabilization on ongoing viral de novo translation.

### G4-3 structural integrity mediates BRACO-19-dependent translational repression

**Fig. 4:**
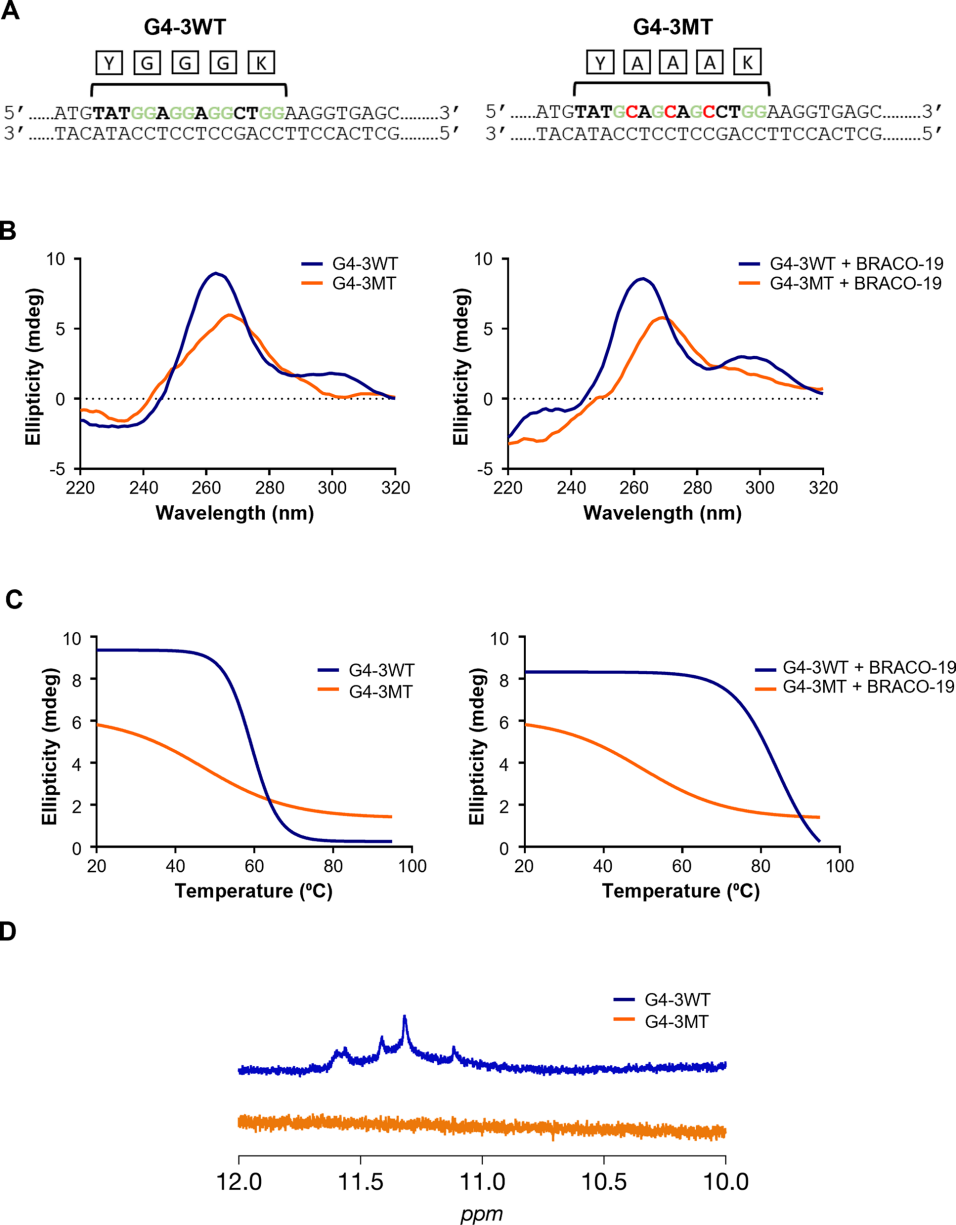

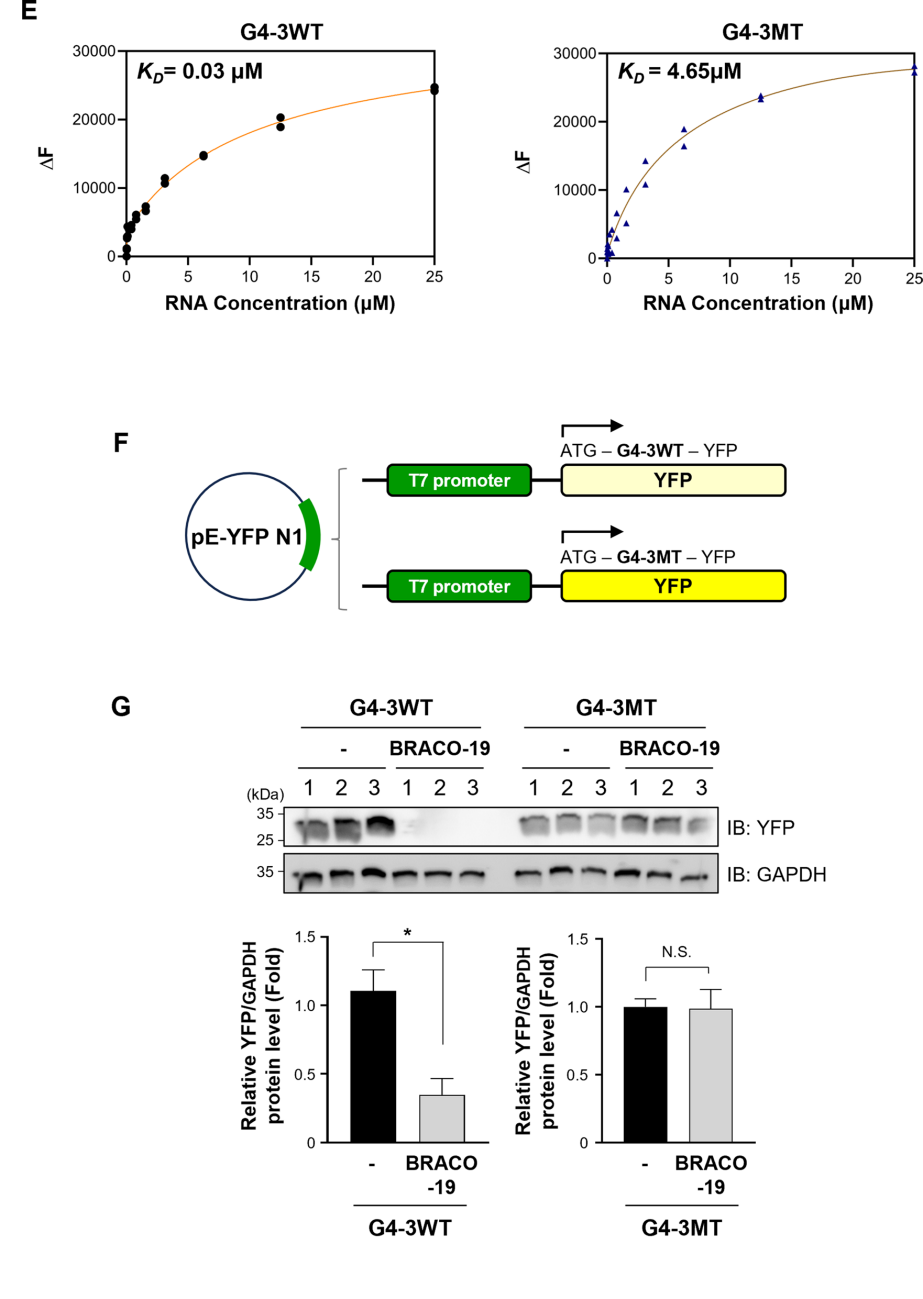
Structural validation of G4-3 links BRACO-19 binding to translational repression. **a** Schematic of the point mutations (marked in red) introduced in the G4-3 sequence (marked in green). **b** CD spectra comparing G4-3WT and G4-3MT show that mutant G4-3 does not form the G4 structure. **c** T_m_ melting analysis showed that BRACO-19 treatment induced only a small T_m_ increase of 2.81 °C in G4-3MT, whereas the G4-3WT exhibited a pronounced stabilization with a T_m_ shift of 25.02 °C. **d** NMR spectra of G4-3WT (blue line): 450 µM of RNA G4s were pre-formed in 10 mM Tris–HCl (pH 7.5) and 100 mM KCl buffer. ^1^H NMR spectra analysis of G4-3 showing the signature imino proton peaks in between the region 11–12 ppm, thus confirming the Hoogsteen bond formation in GQ structure. Absence of these peaks in G4-3MT (orange line) confirms that G4 structure is not formed in the mutant. **e** Fluorescence titration study, the solid, colored lines represent the data plot obtained due to the interaction of BRACO-19 to G4-3WT (Left) sequence and G4-3MT (Right). **f** Schematic representation of pE-YFPN1 reporter plasmid construct with G4-3WT and G4-3MT. **g** IB of expression of YFP using IVT assay for G4-3WT and G4-3MT in the presence and absence of 10 µM BRACO-19. GAPDH was presented as internal loading control

Although several G4 elements exhibited translational regulatory activity (Fig. 3d), G4-3 was selected for further analysis based on a combination of structural stability, ligand responsiveness, and genomic context. While G4-2 shows slightly higher overall sequence conservation than G4-3 (78.6% vs 75.0%), both elements exhibit complete conservation of the G-tract motifs required for G-quadruplex formation. Thus, conservation at the structural motif level is comparable between these candidates. Notably, G4-3 displayed pronounced thermal stabilization upon BRACO-19 binding (Table 1) and robust translational repression (Fig. 3). In addition, its location within the NS3 coding region, which contains a single G4 element, allows more direct functional interpretation without potential interference from adjacent G4 structures. Therefore, we hypothesized that G4-3 plays a major role in BRACO-19-dependent inhibition of viral replication via translation suppression. To validate this hypothesis, a mutant variant (G4-3MT) was generated substituting guanine residues with cytosines to disrupt G4 formation (Fig. 4a). CD spectroscopy and melting analysis confirmed loss of G4 folding, with a reduced T_m_ of 47.09 °C (Fig. 4b, c), suggesting that the folding of G4-3 highly contributes to structural stability. Consistently, the 1H NMR spectrum of the wild-type G4-3 showed distinct imino protein resonances at 11–12 ppm, which is characteristic of Hoogsteen base pairing. By contrast, the mutant G4-3 displayed no imino signals (Fig. 4d), indicating the absence of Hoogsteen base pairing. We next assessed BRACO-19 binding using fluorescence titration, exploiting the intrinsic fluorescence of the ligand as a spectroscopic readout [56]. BRACO-19 bound with high affinity to G4-3 (K_*D*_ = 0.03 μM), whereas its affinity for G4-3MT was markedly reduced (K_*D*_ = 4.65 μM), confirming the G4-specific nature of this interaction between G4-3 and BRACO-19 (Fig. 4e). To further support these structural interpretations, we constructed a reporter containing G4-3MT (Fig. 4f) and compared the translational inhibitory activity of BRACO-19 on the wild-type and mutant reporters. From this assay, we confirmed that BRACO-19 selectively suppressed reporter expression in the presence of an intact G4-3, whereas the mutant construct was unaffected (Fig. 4g). Collectively, these results identify G4-3 as the principal BRACO-19 target and directly link its structural stabilization to translational inhibition.

### Structural characterization of BRACO-19 binding to G4-3

**Fig. 5:**
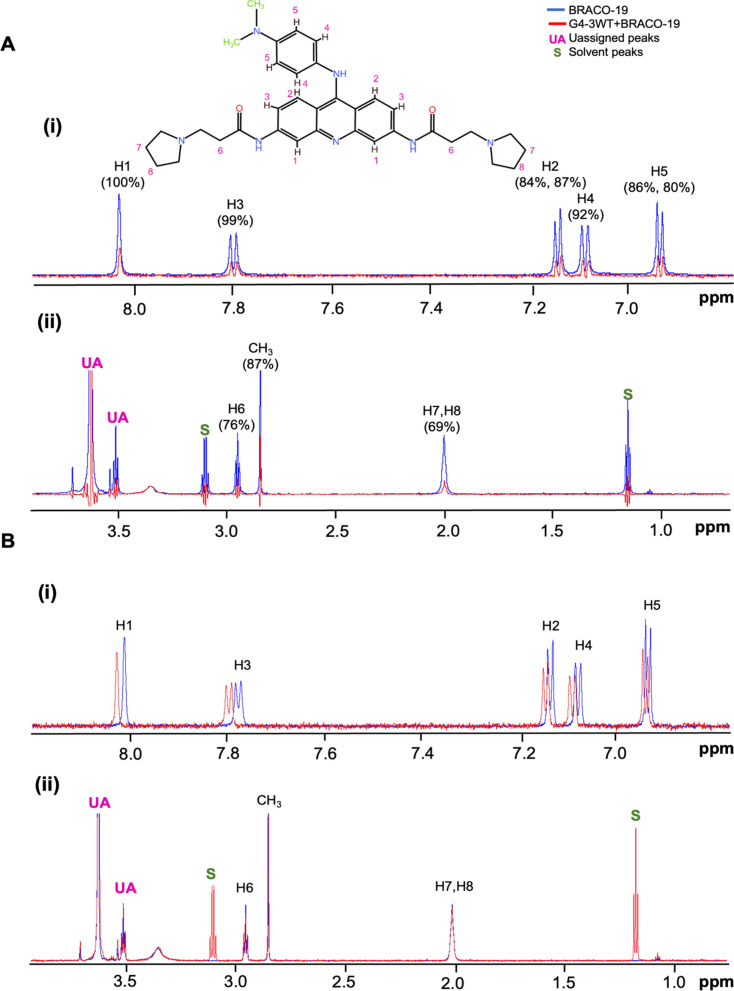

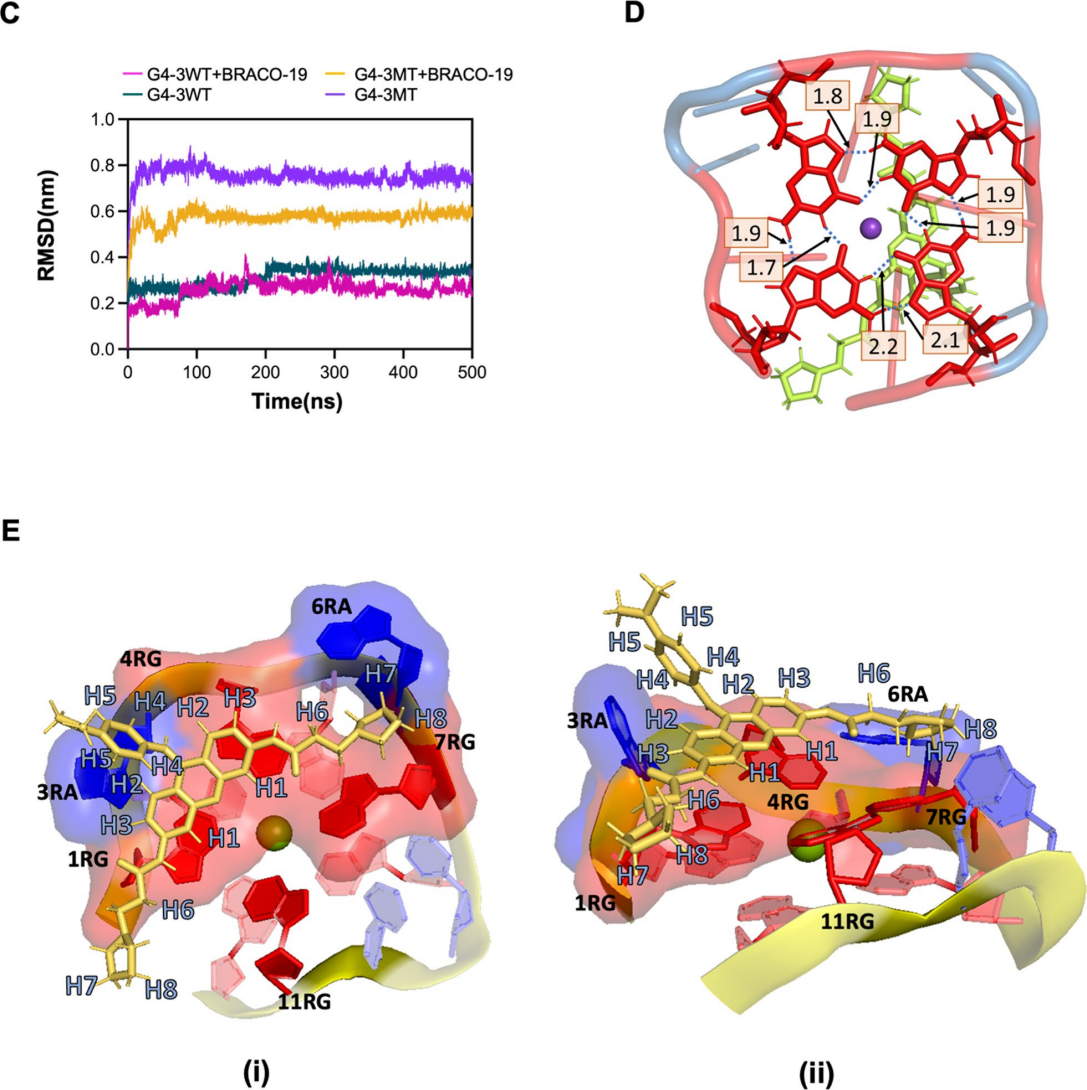
Structural and biophysical characterization of BRACO‑19 binding to the G4‑3. **a** Saturation transfer difference (STD)-NMR analysis of BRACO-19 in the presence of G4-3WT. Comparison of spectra acquired in the absence (blue) and presence (red) of RNA shows intensity reduction in both aromatic (upper panel; H1-H5) and aliphatic (lower panel: H6-H8) proton regions. These results indicate that multiple regions of BRACO-19 are in close proximity to the RNA in the bound state. The percentages shown for each proton represent the relative STD intensity, normalized to the strongest STD signal, which is set to 100%. **b** Chemical shift perturbation (CSP) analysis of BRACO-19 upon binding to G4-3WT. Overlay of ^1^H NMR spectra of free BRACO-19 (blue) and BRACO-19 in the presence of RNA (red) shows pronounced chemical shift changes in aromatic protons (H1–H5), while aliphatic regions remain largely unchanged, indicating that the aromatic core undergoes the most significant local environmental changes upon binding. **c** RMSD plot of G4-3WT and G4-3MT, with and without BRACO-19, showing G4-3 stabilization on BRACO-19 binding throughout 500 ns of production MD run. **d** Intramolecular distances in Å between guanine residues in G4-tetrad after 500 ns simulation with BRACO-19 (Guanine residues in red, BRACO-19 in lime green and K^+^ in purple). **e** Figure depicting 3D molecular interaction map showing the binding environment of BRACO-19 with G4-3 from (i) top view (ii) side view. The ligand (yellow) is surrounded by residues labelled by position 1RG, 3RA, 4RG, 6RA, 7RG, 11RG (1RG denotes the first guanine residue, 3RA denotes third adenine residue, so on and so forth), alongside NMR-derived proton assignments H1-H8. The translucent surface overlay represents the residues interacting with ligand

To directly probe the interaction between BRACO-19 and G4-3 at the molecular level, we performed additional NMR analyses, including saturation transfer difference (STD) and chemical shift perturbation (CSP) experiments. STD responses were detected across both aromatic and side-chain regions, including H6 and the overlapped H7/H8 signals, suggesting that multiple parts of the ligand are positioned in close proximity to the RNA in the bound state (Fig. 5a). In contrast, CSP signals were predominantly observed in the aromatic protons (H1–H5) of BRACO-19, indicating that the aromatic core undergoes significant local environmental changes upon binding (Fig. 5b). These results support a multi-contact binding mode in which the aromatic core and side chains of BRACO-19 contribute differently to the interaction. To further characterize the binding of BRACO-19 to G4-3, we generated 3D models of both the wild-type G4-3 (G4-3WT) and a mutant (G4-3MT) form and subjected them to 500-ns MD simulations in the presence or absence of BRACO-19. The G4-3WT model, constructed using 3D-Nus with hybrid topology inferred from CD spectra, remained largely stable during simulations, whereas G4-3MT was highly unstable, as reflected in elevated RMSD values (Fig. 5c, Fig. S9).In simulations with BRACO-19, G4-3WT maintained stable guanine tetrads coordinate by K⁺ ion (Fig. S9B) (1.7–2.2 Å inter-guanine distance; Fig. 5d), with the ligand adopting an end-stacked binding mode, consistent with BRACO-19 binding modes as previously reported for other G4 [5]. Notably, BRACO-19 was observed to interact not only with the G-tetrad but also with loop residues, indicating a multi-contact binding configuration (Fig. 5e). This binding mode is consistent with the NMR observations, where the aromatic protons exhibit pronounced CSP changes while broader STD responses indicate additional proximity of the side chains (Fig. 5a, b). In contrast, G4-3MT rapidly destabilized regardless of ligand presence, undergoing substantial structural fluctuations (Fig. 5c, Fig. S9C, and S9D). Binding energy calculations further supported this distinction, revealing strong interactions between BRACO-19 and G4-3WT (− 34.79 ± 2.53 kcal/mol) compared with G4-3MT (− 13.67 ± 4.03 kcal/mol).

To gain a structural understanding of the enhanced stabilizing effect and selectivity of BRACO-19 toward the G4-3 structure, we performed comparative molecular modeling and molecular dynamics (MD) simulations using two representative G4-binding ligands, PhenDC3 and Quercetin (Fig. S10). PhenDC3 is a well-established high-affinity G4 ligand, whereas Quercetin represents a comparatively weaker and less selective binder. Consistent with this distinction, CD spectroscopy and thermal melting analyses showed that Quercetin induces only modest structural changes and limited thermal stabilization of G4-3 compared to BRACO-19 (Fig. S4 and Fig. S5). Binding free energy calculations revealed that PhenDC3 exhibits a binding free energy (− 34.71 ± 3.10 kcal/mol) comparable to that of BRACO-19, whereas Quercetin shows a significantly weaker binding affinity (− 22.52 ± 3.66 kcal/mol). Structural analyses further revealed clear differences in binding modes among the ligands. PhenDC3 predominantly interacts with the guanine tetrad core (Fig. S10B, S10E), suggesting a tetrad-centered binding mode. In contrast, Quercetin adopts a more localized interaction pattern, primarily engaging loop residues, particularly around 10RU, with limited and less stable contacts with the G-tetrad core (Fig. S10C and S10F). Notably, BRACO-19 establishes more extensive and distributed interactions, simultaneously engaging both the G-tetrads and loop regions (Fig. S10A and Fig. S10D). This dual interaction mode, involving end-stacking on the G-tetrad core and additional contacts with loop residues, likely contributes to enhanced stabilization and structural selectivity of the G4-3 complex. Furthermore, RMSD and RMSF analyses indicate that all ligand-bound systems maintain overall G4 structural integrity during the simulation (Fig. S10G and S10H). However, BRACO-19 supports a more stable interaction network while preserving the rigidity of the G-tetrad core, whereas Quercetin exhibits more localized interactions accompanied by greater flexibility, particularly in loop regions. PhenDC3 maintains stable tetrad interactions but shows limited engagement beyond the core structure. Collectively, these findings demonstrate that, despite comparable binding energies in some cases (e.g., PhenDC3), the mode of interaction, particularly the ability to engage both tetrad and loop elements, plays a critical role in determining effective stabilization and selective targeting of the viral RNA G-quadruplex by BRACO-19.

### BRACO-19 targets the conserved G4-3 RNA G-quadruplex to suppress DENV translation

**Fig. 6:**
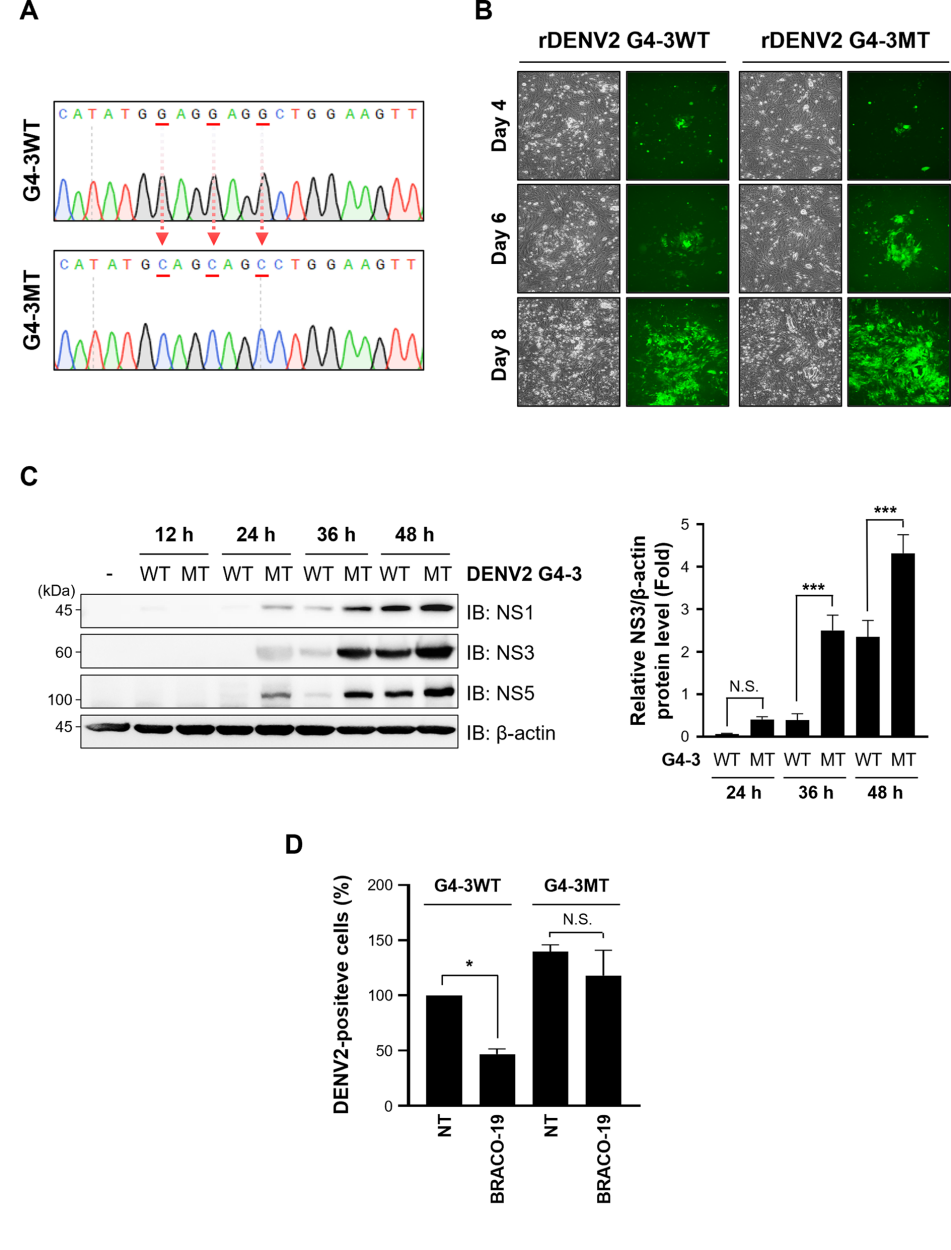

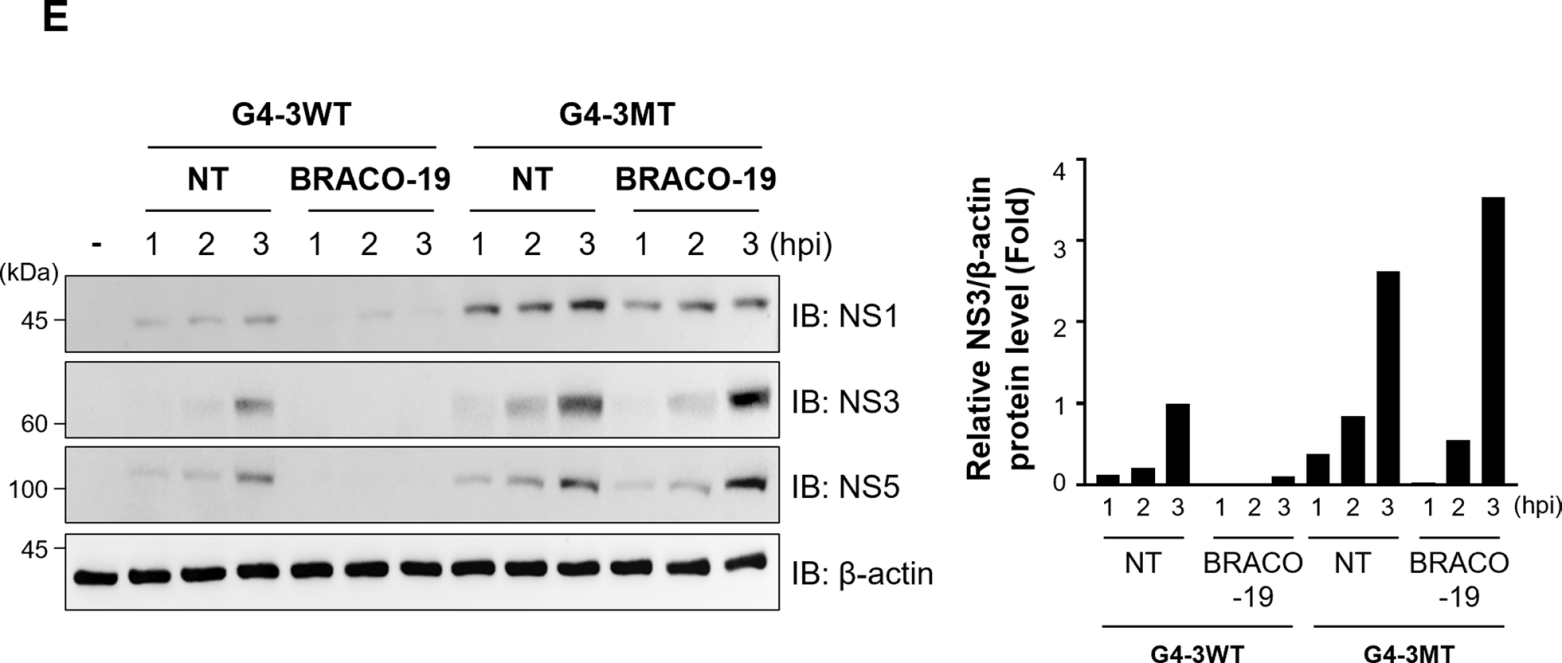
The G4-3 RNA G-quadruplex governs BRACO-19-mediated inhibition of DENV2 translation. **a** Construction of recombinant DENV genome containing G4-3MT via site-directed mutagenesis and sequencing of G4-3WT and G4-3MT.** b** Generation of DENV2 recombinant virus encoding G4-3WT and MT that contain GFP reporter gene. 293FT was transfected with CPER product and co-cultured with Vero76. Spreading of GFP means viral propagation.** c** Comparison of viral gene expression level of G4-3WT and G4-3MT viruses. Vero76 was infected with G4-3WT or G4-3MT MOI 1.0 for indicated timepoint. (Left) NS1, NS3, and NS5 were detected by IB. (Right) Relative protein expression level of NS3 and β-actin was measured by ImageJ.** d** Sensitivity of BRACO-19 against G4-3WT and G4-3MT. Vero76 was infected with MOI 1.0 of G4-3WT or G4-3MT and treated with 25 µM of BRACO-19 for 48 h. DENV2-positive cells were then detected by flow cytometry. **e** Translational inhibition of DENV2 by BRACO-19. Vero76 was infected with MOI 5 of G4-3WT or G4-3MT, and viral proteins were detected at the indicated timepoint. (Left) NS1, NS3, and NS5 were detected by IB. (Right) Relative protein expression level of NS3 and β-actin was measured by ImageJ. P-value was calculated by One-way ANOVA and multiple comparison (N.S., *p* > 0.05; *p < 0.05; ***p < 0.001)

To evaluate the functional contribution of G4-3 to DENV replication, we generated recombinant GFP-expressing DENV2 containing either the G4-3WT or G4-3MT version of the G4-3 sequence (Fig. 6a). The full-length viral genome was divided into eight overlapping fragments, each PCR-amplified form from plasmid templates, and reconstituted by circular polymerase extension reaction (CPER). In this strategy, either the intact or mutant G4-3 sequence was specifically introduced into the fragment encompassing the native G4-3 region. Transfection of CPER products into 293FT cells, followed by co-culture with Vero76 cells, enabled efficient viral rescue, as confirmed by GFP expression from both G4-3WT and G4-3MT recombinant viruses (Fig. 6b). After quantifying and normalizing viral titers, equal amounts of G4-3WT and G4-3MT recombinant viruses were used to infect Vero76 cells (Table S5), and viral replication was monitored by IB for DENV proteins. Compared to the G4-3WT virus, the G4-3MT virus consistently exhibited elevated protein expression and higher replication capacity, indicating that disruption of the G4-3 structure provides a replication advantage (Fig. 6c). We next examined the responsiveness of these recombinant viruses to BRACO-19. As expected, BRACO-19 had no measurable effect on the mutant virus, whereas it markedly inhibited the replication of G4-3WT (Fig. 6d). Consistent with this result, recombinant G4-3MT DENV enhanced viral gene expression and reduced susceptibility to BRACO-19 (Fig. 6e). This striking differential response demonstrates that the antiviral activity of BRACO-19 is strictly dependent on an intact G4-3 element. Taken together, these results establish G4-3 as a critical regulatory element in the DENV2 genome and identify it as the principal target through which BRACO-19 mediates its antiviral activity.

### BRACO-19 showed significant antiviral efficacy against DENV2 in an in vivo AG129 mouse model

**Fig. 7:**
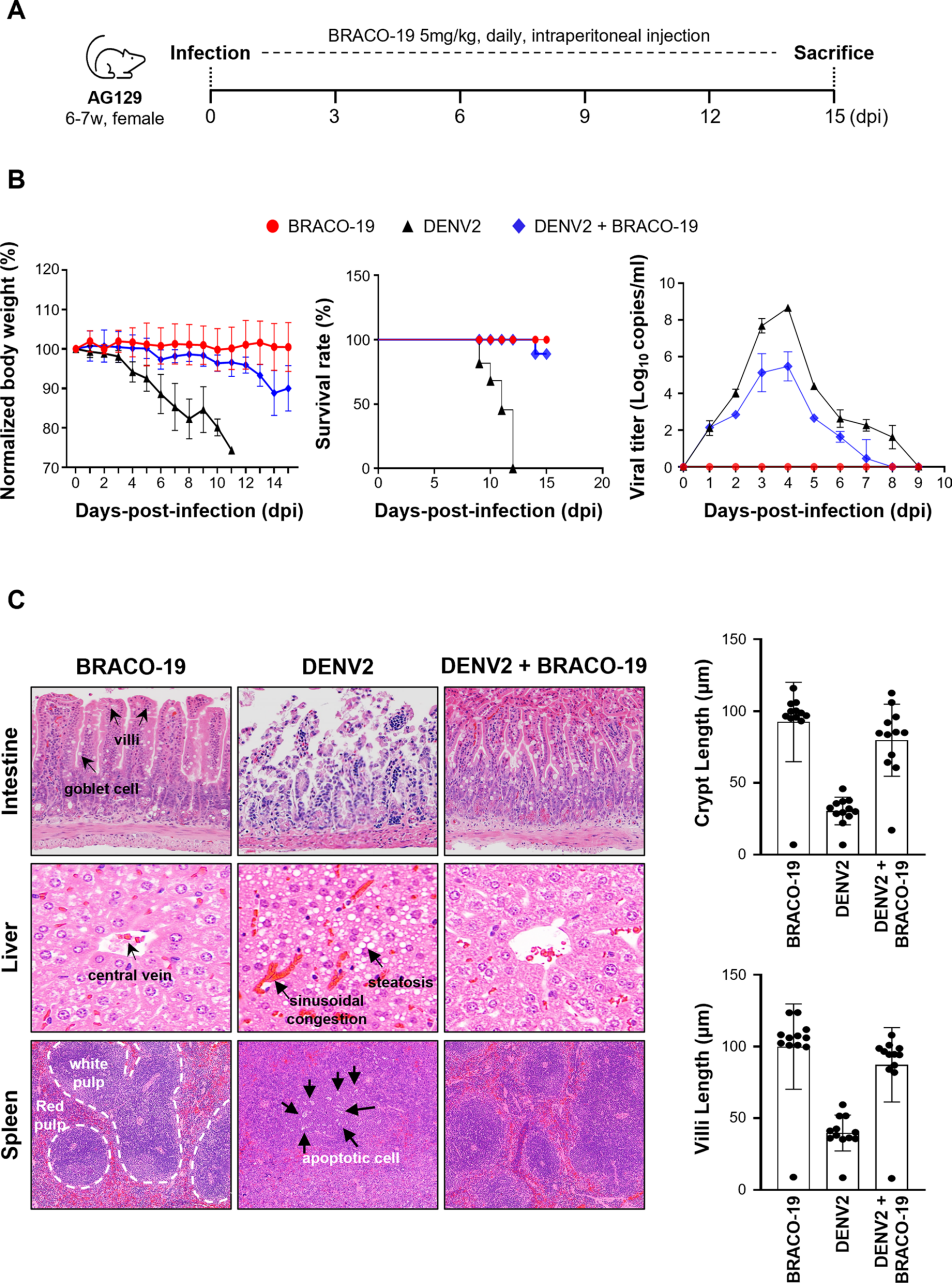
BRACO-19 treatment exerts therapeutic efficacy against DENV2 infection in AG129 mice. **a** Schematic of BRACO-19 treatment against DENV2-infected mouse experiment. AG129 was infected with DENV2 (1 × 10^6^ FFU/mouse) and treated with BRACO-19 (5 mg/kg) daily by intraperitoneal injection (BRACO-19; n = 5, DENV2; n = 5, DENV2 + BRACO-19; n = 5). **b** Body weight (Left), Kaplan–Meier survival curve (Middle), and viral RNA copy numbers in serum (Right) of each mouse group. **c** Histological analysis of tissues from DENV2-infected AG129 after sacrificing at 15 dpi via hematoxylin and eosin (H&E) staining. Histopathological changes were evaluated by quantitative morphometric analysis of intestinal crypt length and villus length (μm) in each group

We next evaluated the in vivo efficacy of BRACO-19 against DENV2 infection using 6–7-week-old female AG129 mice (n = 5 mice per group). Animals were intraperitoneally (IP) challenged with a lethal dosage of 1 × 10^6^ FFU DENV2 and subsequently treated with BRACO-19 (5 mg/kg, IP, once daily for two weeks) (Fig. 7a). DENV2-infected mice receiving BRACO-19 exhibited a transient early weight loss that subsequently recovered, whereas untreated infected mice showed continuous weight loss. Importantly, all mice in the DENV2-only group succumbed to infection, while 4 of 5 infected mice treated with BRACO-19 survived (Fig. 7b).

Mice treated with BRACO-19 alone displayed no weight loss, mortality, or detectable signs of toxicity. Consistent with this outcome, BRACO-19 treatment significantly reduced viremia (Fig. 7b). Further confirmation was obtained through histopathological analysis of major organs. In the H&E staining results, lipid droplet accumulation was observed within the cytoplasm of hepatocytes in the DENV2-infected group, whereas neither lesion was detected in the BRACO-19-treated group, marked loss of mucosal layer structure and necrotic findings were observed in the small intestine. In contrast, both the BRACO-19-treated group and the BRACO-19 plus DENV2 co-administered group showed intact villi-crypt architecture. In the spleen, the DENV2-infected group showed no change in red pulp area or hematopoietic density compared with the control group, but hyperplasia of the white pulp was observed, and the boundary between the germinal center and marginal zone became poorly defined. In contrast, BRACO-19 treatment largely preserved normal splenic follicle architecture. Mice co-administered DENV2 and BRACO-19 displayed mild to moderate white pulp hyperplasia. However, proliferation was notably less prominent than in the DENV-infected group (Fig. 7c). Collectively, these results demonstrate that BRACO-19 confers significant efficacy in vivo against DENV2 infection.

## Discussion

The high mutation rate of RNA viruses continues to undermine conventional antivirals against DENV, including nucleoside analogues and protein-targeted inhibitors. These limitations underscore the need for strategies that engage conserved genomic structures rather than mutation-prone viral proteins. In this context, our findings support the emerging concept of targeting structured nucleic acid elements formed by highly conserved genomic sequences. In particular, RNA G4 represents an appealing class of such targets adopt defined three-dimensional conformations that remain stable even in the presence of single- or double-nucleotide substitutions, making them attractive and resilient antiviral targets [30]. Although previous works identified putative G4 motifs in the DENV genome [53], their precise functional relevance and potential as druggable sites remain unclear.

Here, we provide a comprehensive, mechanism-based framework for G4-targeted antiviral intervention. To assess how G4 binding relates to antiviral activity, we employed a panel of well-characterized ligands spanning diverse chemotypes binding modes. Acridine-based compounds (BRACO-19 and PhenDC3) exhibit high-affinity G4 stabilizing, whereas TMPyP4 primarily via external stacking and its non-planar isomer TMPyP2 serves as a low-activity control [40, 45, 66]. Structurally distinct ligands, including thioflavin T, quercetin, and pyridostatin (PDS), were also included to capture variation in scaffolds and selectivity [4, 19, 27]. This approach enabled direct linkage of antiviral activity to ligand-specific G4-binding properties (Fig. 2a). To identify the structural targets underlying these effects, we performed systematic biophysical screening of candidate G4s. Among the seven candidates G4s, G4-3 emerged as the most structurally stable and highly conserved element across all four serotypes. BRACO-19 strongly stabilized this element with nanomolar binding affinity, resulting in pronounced antiviral activity across multiplicities of infection (MOIs) and serotypes, along with a favorable therapeutic index. Mechanistically, stabilization of G4-3 by BRACO-19 selectively suppresses viral translation rather than transcription, consistent with dependence of DENV on immediate translation of its positive sense RNA genome. Genetic validation further confirmed the functional importance of this element: recombinant DENV2 harboring a G4-disruptive mutation (rDENV2-G4-3MT) exhibited enhanced replication and reduced sensitivity to BRACO-19 compared with the wild-type virus. Together, these results demonstrate that the antiviral activity of BRACO-19 is dependent on the structural integrity of G4-3.

RNA G4s are known to regulate translation through position-dependent manner. While G4 structures located in the 5′UTR can interfere with ribosome scanning during translation initiation, G4s positioned within coding regions have been reported to impede ribosome progression, leading to translational pausing or stalling. Consistent with this, previous studies have shown that increased G4 stability correlates with reduced translational output [12]. In this study, G4-3 is located within the NS3 coding region, suggesting that its stabilization by BRACO-19 likely interferes with ribosome progression rather than initiation. This interpretation is supported by the reporter assays demonstrating that translational repression is dependent on the intact G4 structure and is abolished upon disruption of the G4 motif. Together, these findings support a model in which ligand-induced stabilization of RNA G4 structures inhibits viral protein synthesis by impeding ribosome movement.

Our translation “chase” experiments further clarify the mode of action of BRACO-19 (Fig. S6). The time-dependent effects suggest that its antiviral activity is most pronounced during the early phase of infection, when viral replication relies on de novo translation of the incoming positive-sense RNA genome. According, the reduction in viral RNA levels at early time points is most consistent with a secondary consequence of impaired accumulation of nonstructural proteins, rather than a direct suppression of RNA replication. This interpretation is supported by the minimal antiviral effect observed when BRACO-19 is added at later stages of infection, when sufficient viral components have already accumulated to sustain replication. The lack of inhibition at these stages is inconsistent with mechanisms targeting downstream processes such as minus-strand RNA synthesis or virion assembly. Instead, BRACO-19 obstructs viral translation, thus preventing the accumulation of essential nonstructural proteins, including NS3 and RdRp, and consequently impairing genomic replication and virion assembly. Nonetheless, we cannot entirely exclude additional mechanisms beyond translation. Given that the G4-3 element is located in the NS3 coding region, stabilizing this structure could, in theory, impact processes like RdRp progression or higher-order RNA organization, both of which are crucial for genome packaging and virion assembly. Although these possibilities are not mutually exclusive of the primary translation-based mechanism, they are not directly examined in the current study and warrant further investigation to fully elucidate how G4-3 stabilization contains the DENV life cycle. Based on these mechanistic insights, it is crucial to emphasize the in vivo efficacy of BRACO-19, highlighting its therapeutic potential. BRACO-19 provided significant in vivo protection, diminishing viremia and enhancing survival to 80% in infected AG129 mice, while histopathological examination revealed notable preservation of hepatic, intestinal, and splenic architecture relative to untreated controls [8, 16]. These results collectively address a significant knowledge gap in prior genome-G4 surveys by (i) pinpointing G4-3 as the functionally relevant and evolutionarily conserved genomic element essential for the DENV life cycle, and (ii) defining a translation-centric mechanism of antiviral action that is further supported by CHX-based chase experiments.

Developing small molecules against G4s requires balancing potency, selectivity, and cellular tolerability. Most G4 ligands stabilize targets by π–π stacking on terminal G-tetrads, aided by cationic sidechains that engage grooves/loops; however, limited selectivity over other G4s or duplex nucleic acids has historically impeded clinical translation [14]. In this study, several representative ligands were screened side-by-side, and BRACO-19 showed the strongest stabilization of G4-3 in vitro and the highest antiviral activity in cells, yielding an approximate nine-fold therapeutic window in Vero cells (CC₅₀/IC₅₀ ≈ 158.6/17.74). Mechanistically, prior structural and simulation work indicates that BRACO-19’s acridine core end-stacks on G-tetrads, while protonatable sidechains sample groove/loop contacts can provide both high stabilization and topology tolerance across parallel/hybrid folds [32]. By contrast, PhenDC3 is a bisquinolinium ligand with very high intrinsic G4 affinity and robust stabilization across diverse G4s, properties that make it a powerful chemical probe but also less discriminating in cellular contexts (and often more cytotoxic) [41]. In contrast, Quercetin exhibits significantly weaker binding affinity and limited stabilizing capacity toward G4 structures, primarily interacting with loop regions with minimal engagement of the G-tetrad core. Consistently, structural analyses of the BRACO-19, Quercetin, and PhenDC3 binding model to G4-3WT in this study (Fig. 5 and S10) reveal that BRACO-19 shows consistent interaction with not only the G-tetrad residues but also the loop residues, while PhenDC3 largely interacts with the G-tetrad residues only, and Quercetin displays more localized and less stable interactions, predominantly with loop residues, which likely helps BRACO-19 maintain more stable contacts with G4-3WT as compared to other ligands. Taken together, these binding-mode differences offer a structure-based rationale for why only BRACO-19 among the ligands tested produced the best net profile (strong G4-3 stabilization with acceptable cellular tolerability) in our hands.

Despite these advantages, achieving robust selectivity remains a central challenge. BRACO-19 illustrates both the potential and the limitations of first-generation pan-G4 ligands: its planar acridine core promotes high-affinity π–π stacking with terminal G-tetrads, a binding mode conserved across host and viral G4s, thereby predisposing to off-target engagement. Its polycationic side chains further constrain membrane permeability and contribute to suboptimal ADME–tox profiles. In the context of DENV infection, however, two features substantially alleviate this concern. First, viral RNA accumulates to high copy numbers and becomes spatially concentrated within replication organelles (vesicle packets) [9], thereby elevating the local effective concentration of viral G4 targets relative to widely dispersed host transcripts and DNA. Second, the functional and topological context of G4-3 (embedded within the NS3 coding region) differs markedly from that of most cellular G4s, enabling structure-guided ligand design that exploits sequence, loop, and groove features unique to this element (Fig. 5 and S10). Nonetheless, within this study, BRACO-19 exhibited a favorable therapeutic index and validated DENV G4-3 as a druggable RNA element. Targeting G4 structures may also confer an intrinsic advantage in resistance. Targeting G4 structures may further confer an intrinsic advantage in mitigating resistance. In contrast to nucleoside analogs, which target the viral polymerase and are susceptible to resistance through mutations that alter drug incorporation or excision, G4s represent higher-order RNA structures that can accommodate certain nucleotide variations without loss of folding. This structural resilience may reduce susceptibility to escape via single-point mutations. Future efforts should therefore shift from symmetric tetrad-stacking scaffolds toward more diverse, viral-selective chemotypes. Structure-guided design, informed by molecular dynamics, can exploit the distinct loop flexibility and RNA-specific groove architecture of DENV G4-3, reducing planarity and charge while enabling sequence- and topology-specific interactions. Such strategies are expected to enhance selectivity and improve pharmacokinetic profiles.

## Conclusion

In conclusion, this study provides the first evidence that targeting RNA G4 within the DENV genome offers a viable therapeutic strategy. By demonstrating that structural stabilization of these elements disrupts viral translation and suppresses replication across all DENV serotypes, we establish a strong proof of concept for RNA structure–based antiviral intervention. Because many G4 motifs are conserved across flaviviruses, these findings may extend beyond dengue, supporting quadruplex stabilization as a potential broad-spectrum antiviral approach. Integrating RNA structural biology with rational ligand design could thus pave the way for next-generation antiviral capable of overcoming the evolutionary resilience of RNA viruses. Despite the promising selectivity of BRACO-19 for the G4-3 element, further development will require systematic evaluation of drug-like properties and off-target effects. Nevertheless, the specificity revealed here highlights G4-3 within the DENV genome as a tractable antiviral target and may provide a basis for extending G4-directed strategies to other flaviviruses.

## Supplementary Information

Additional file1 (PDF 1968 KB)

## Data Availability

All data generated or analyzed during the current study are available in this published article and its supplementary information files.

## Abbreviations

CC_50_: Half-maximal cytotoxic concentration
CD: Circular dichroism
CPE: Cytopathic effect
CPER: Circular polymerase extension reaction
DENV: Dengue virus
DPI: Days post-infection
G4: G-quadruplex
IB: Immunoblotting
IC_50_: Half-maximal inhibitory concentration
IP: Intraperitoneal
MOI: Multiplicity of infection
NMR: Nuclear magnetic resonance
NS: Non-structural protein
