# Supplementary material for: Targeting a genomic RNA G-quadruplex of dengue virus with small molecules as an alternative to protein-targeted therapeutics
Source: J Biomed Sci. 2026 May 27;33:55. doi: 10.1186/s12929-026-01262-x (PMC13217665; doi:10.1186/s12929-026-01262-x)
Supplement: Supplementary file 1 — Additional file1 (PDF 1968 KB) [file 12929_2026_1262_MOESM1_ESM.pdf]

Supplementary Figures and Tables

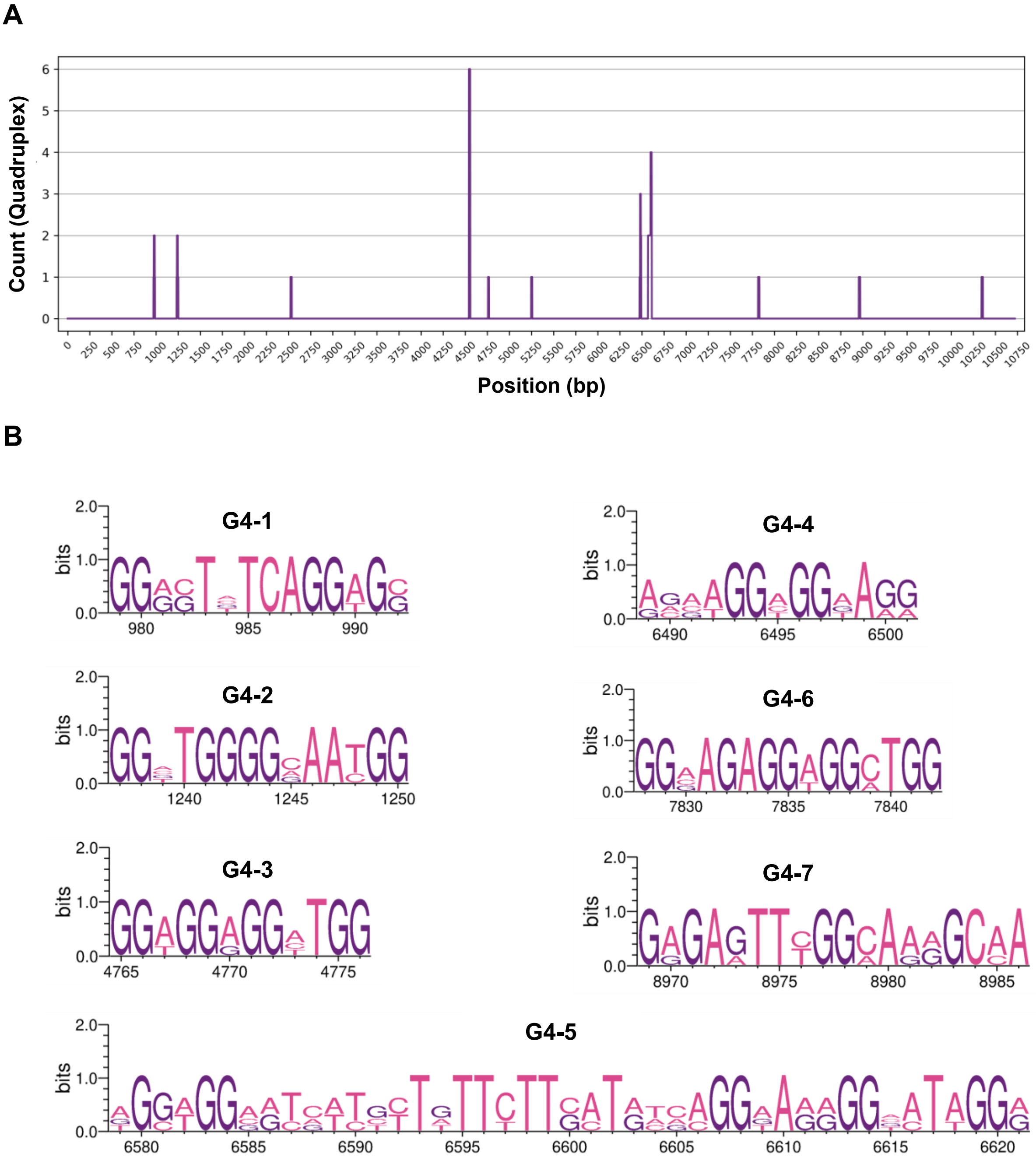

Figure S1. G4 prediction in DENV serotype 2 genome and their conservation across DENV serotypes

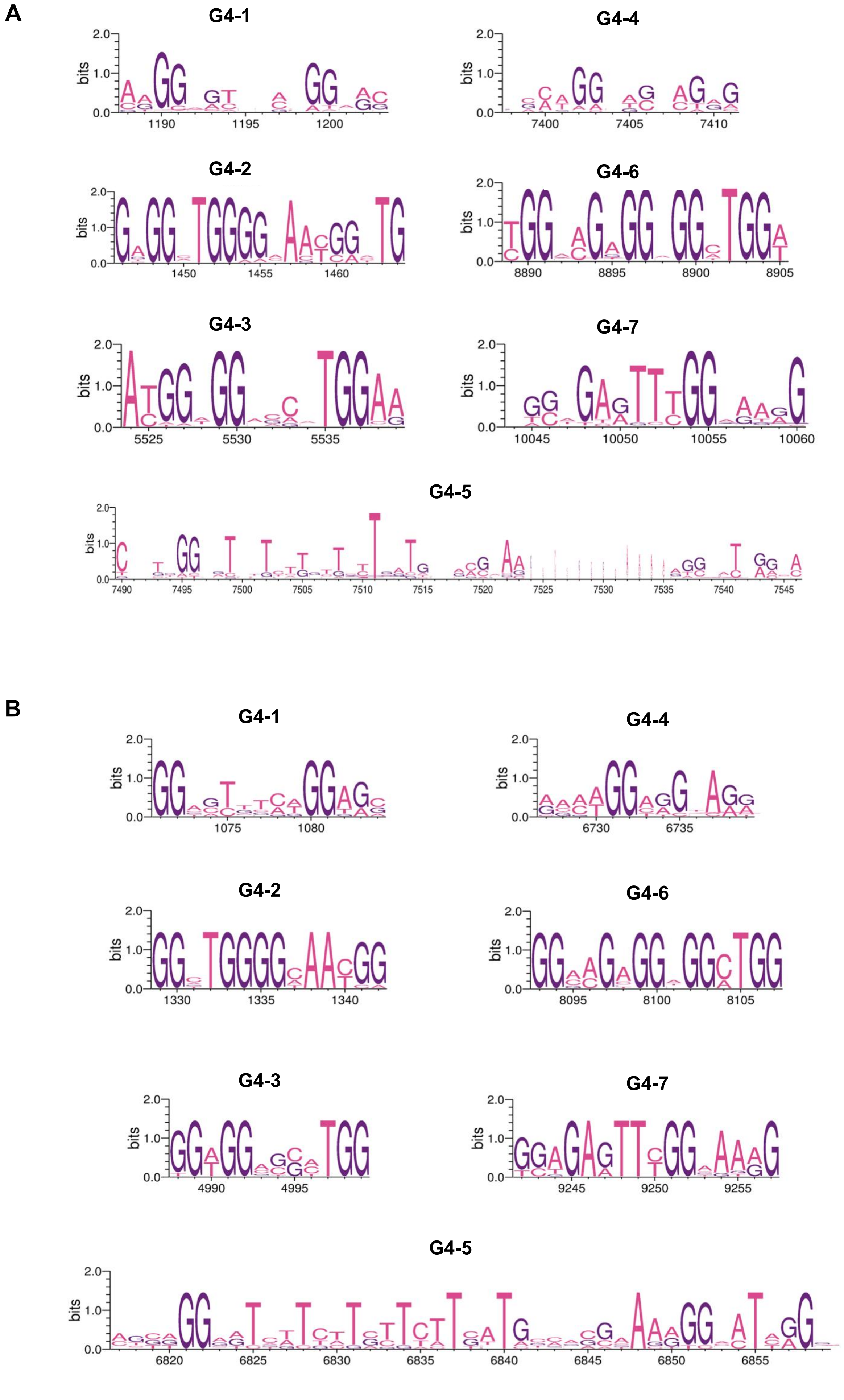

Figure S2. Conservation of G4 motifs across flaviviruses

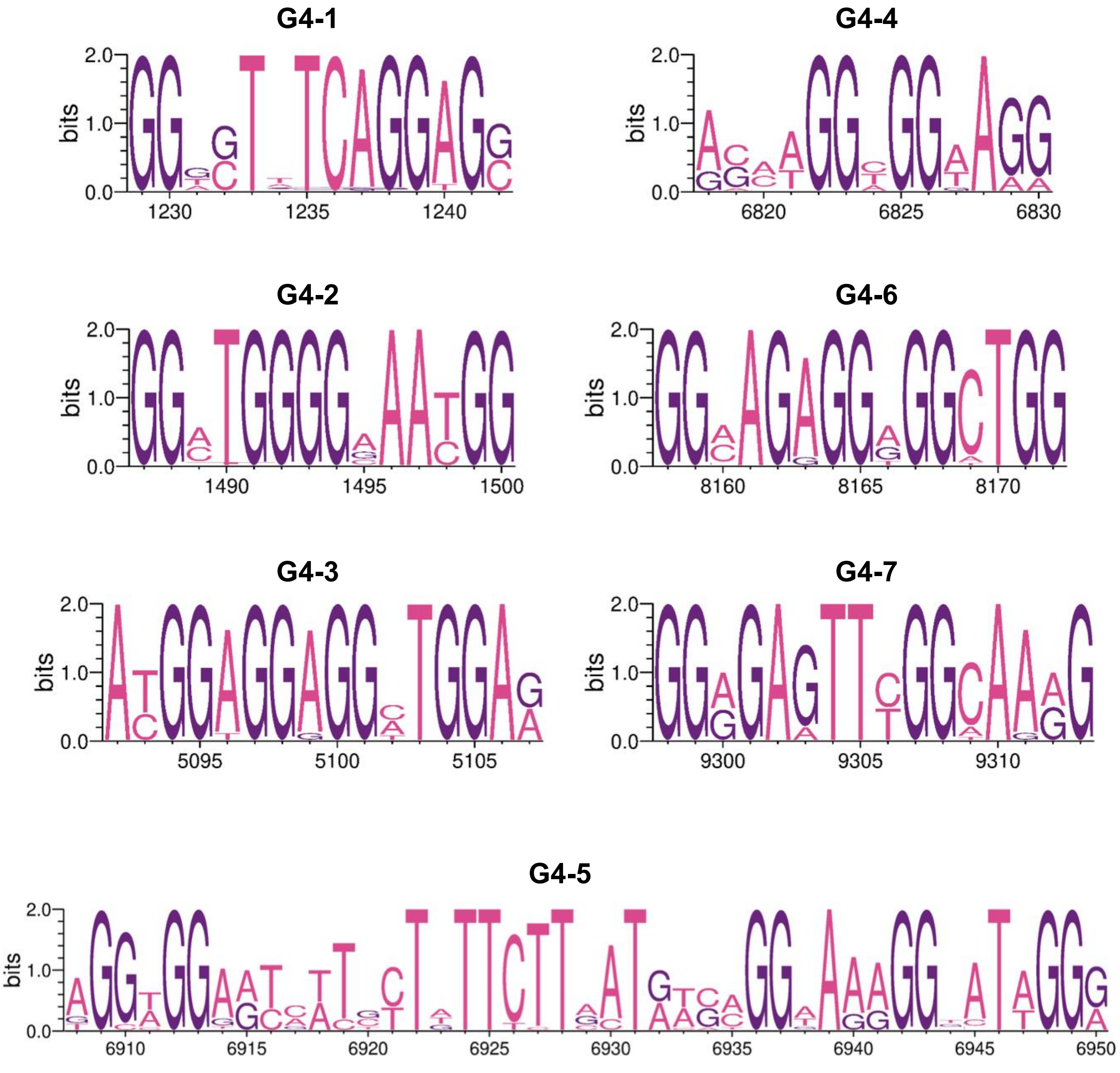

Figure S3. Large-scale analysis of G4 motif conservation across DENV sequences

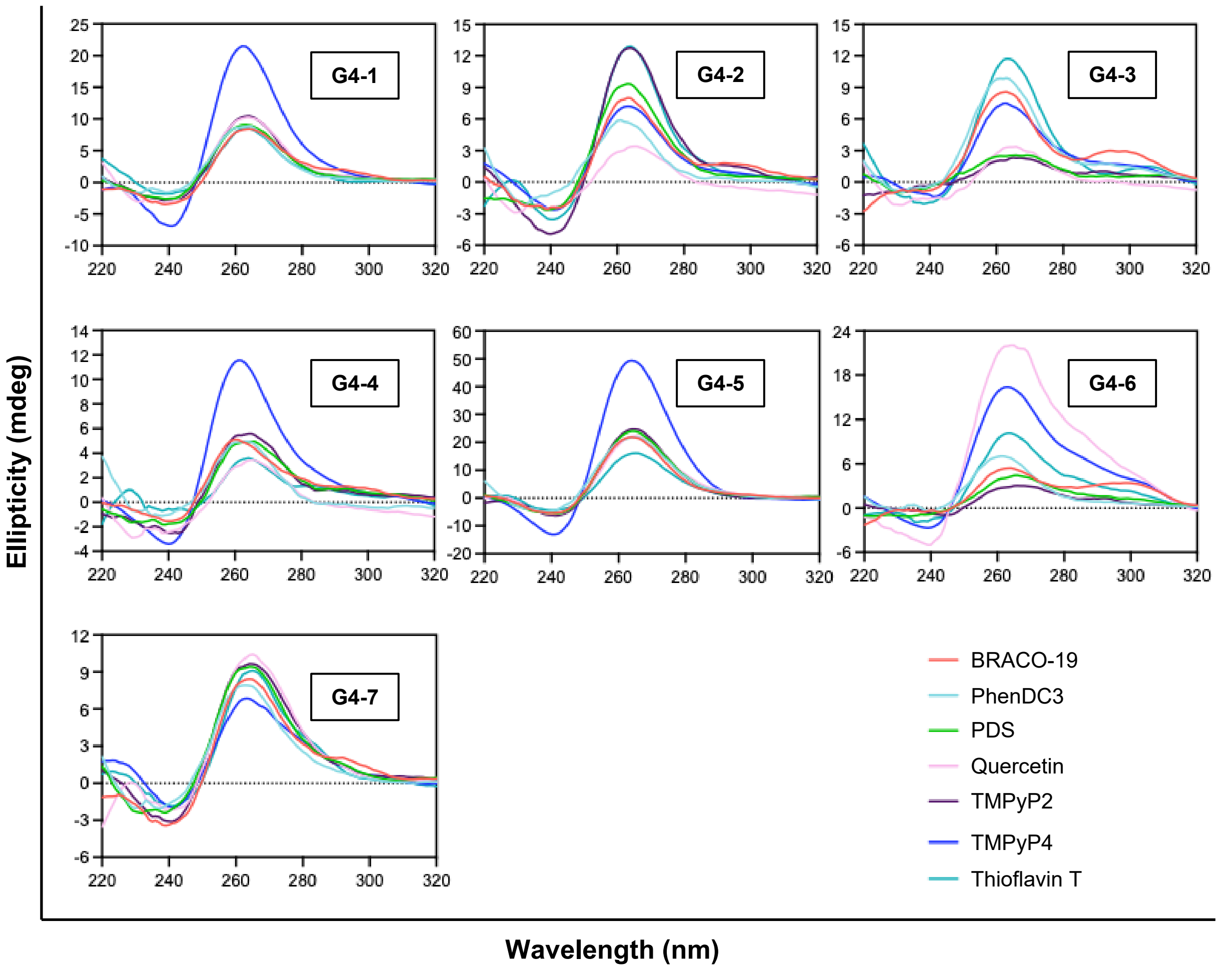

Figure S4. G4 topologies exhibit distinct CD signature

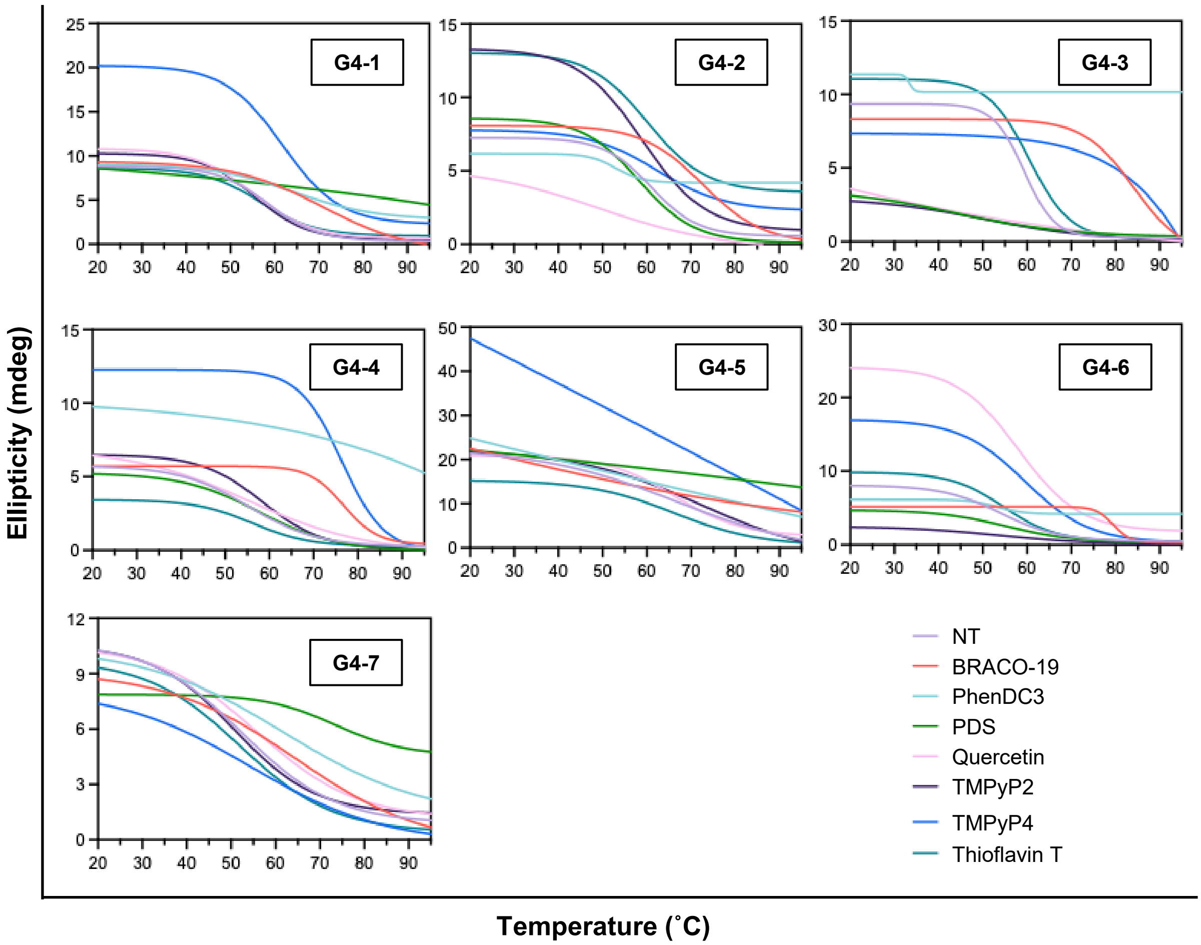

Figure S5. T_m analysis for G4 structures in presence of different G4 ligands

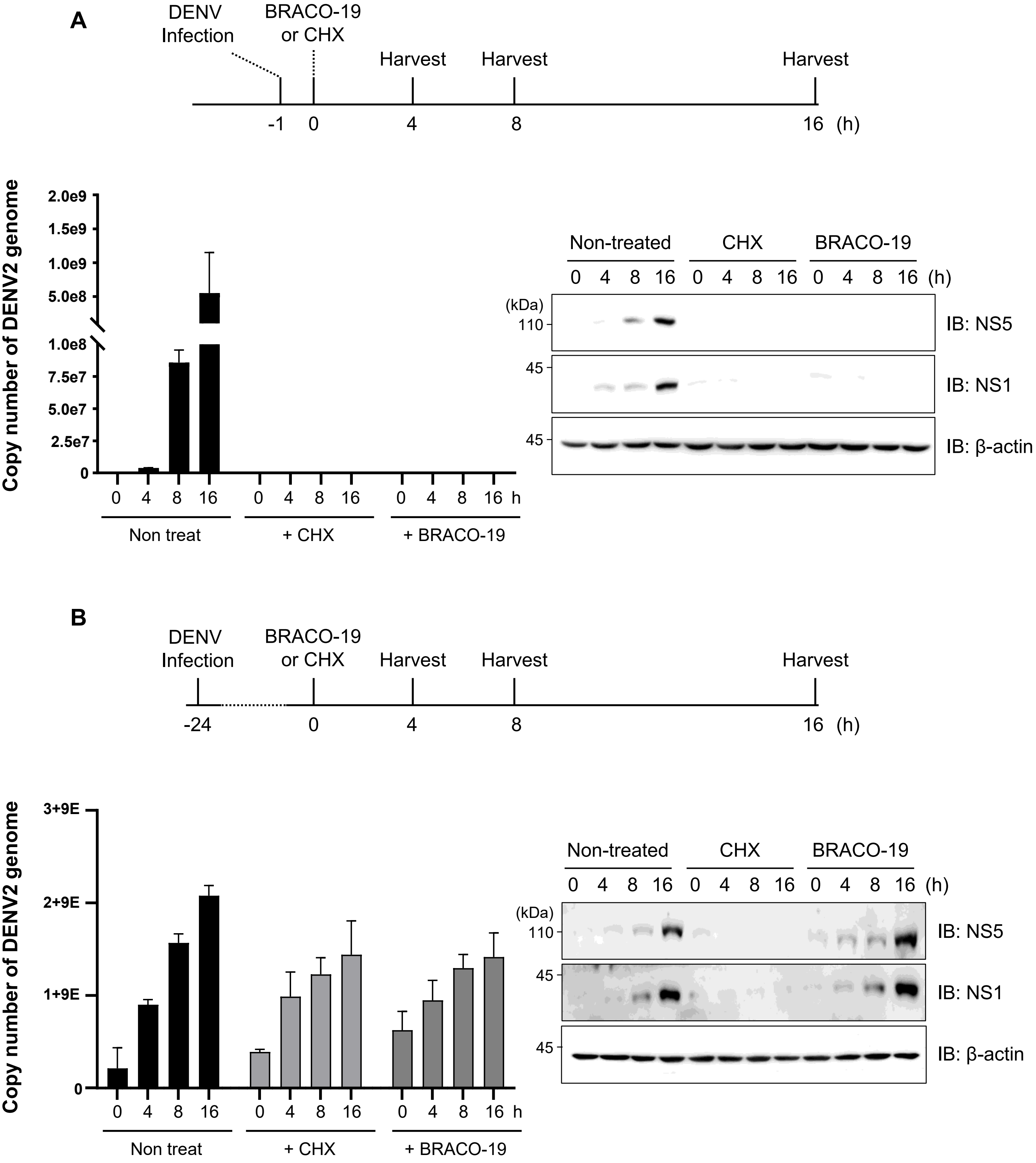

Figure S6. Cycloheximide chase analysis of BRACO-19-mediated translational inhibition of DENV2

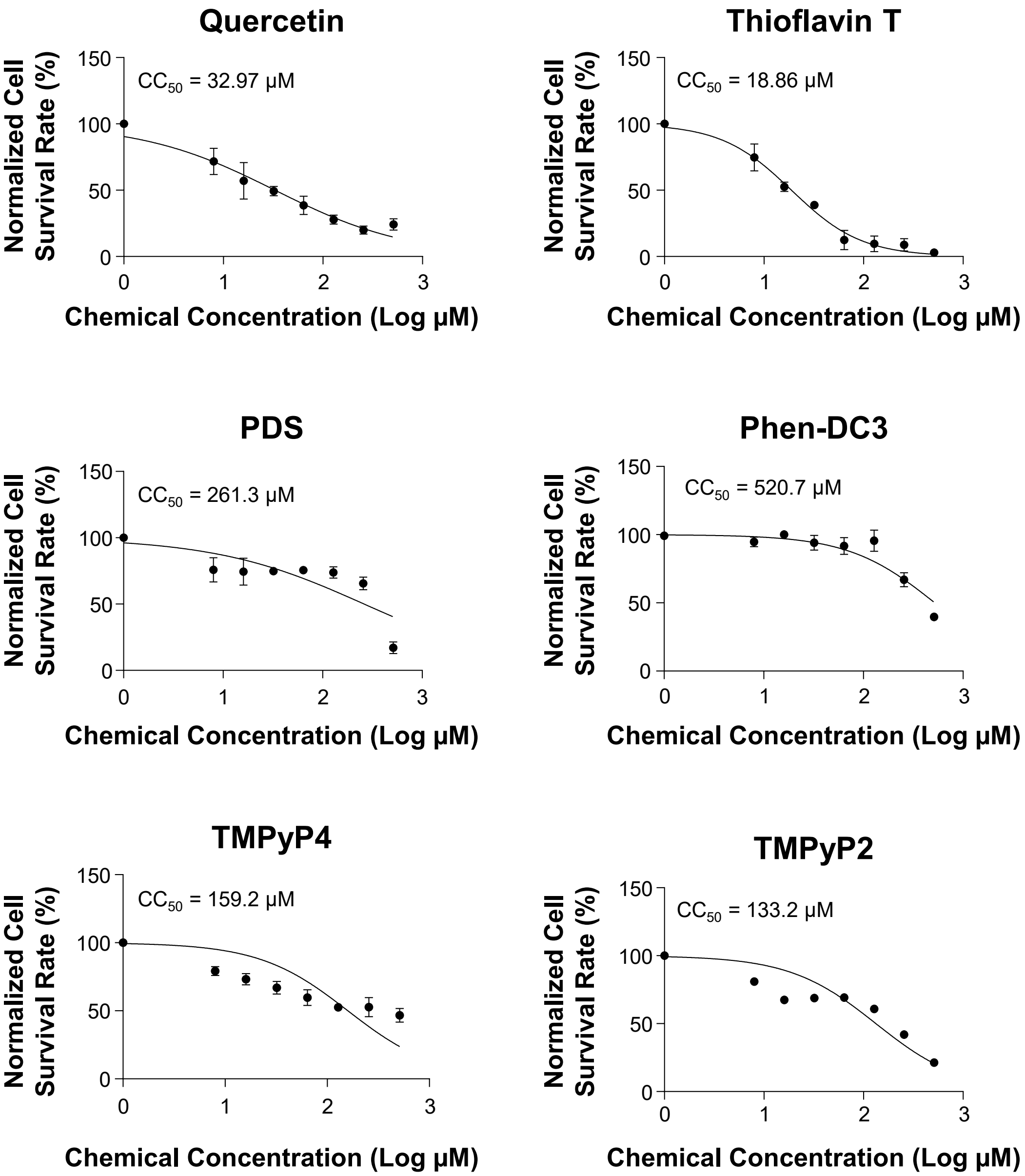

Figure S7. The cytotoxicity profile of G4 ligands

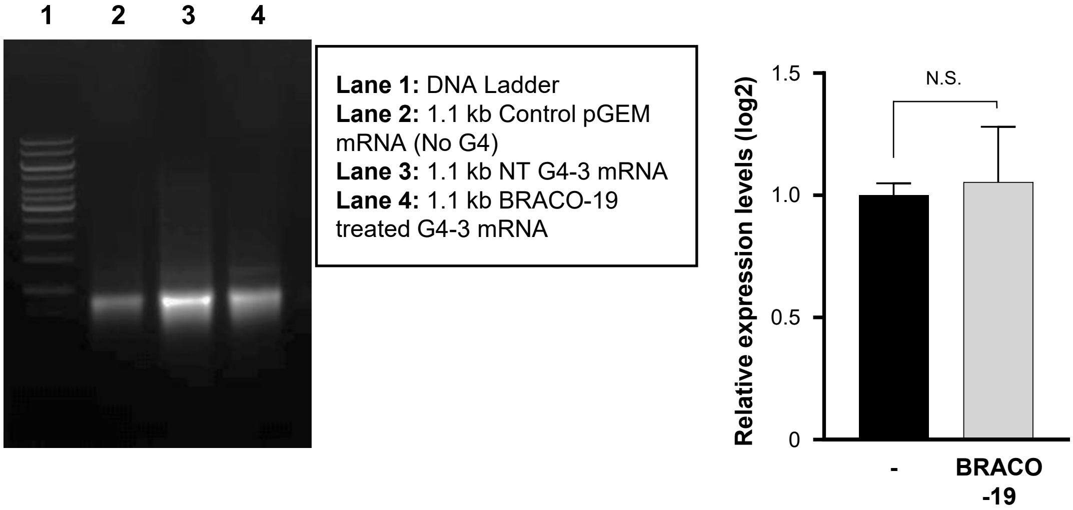

Figure S8. The effect of BRACO-19 on the transcription of the G4-containging reporter

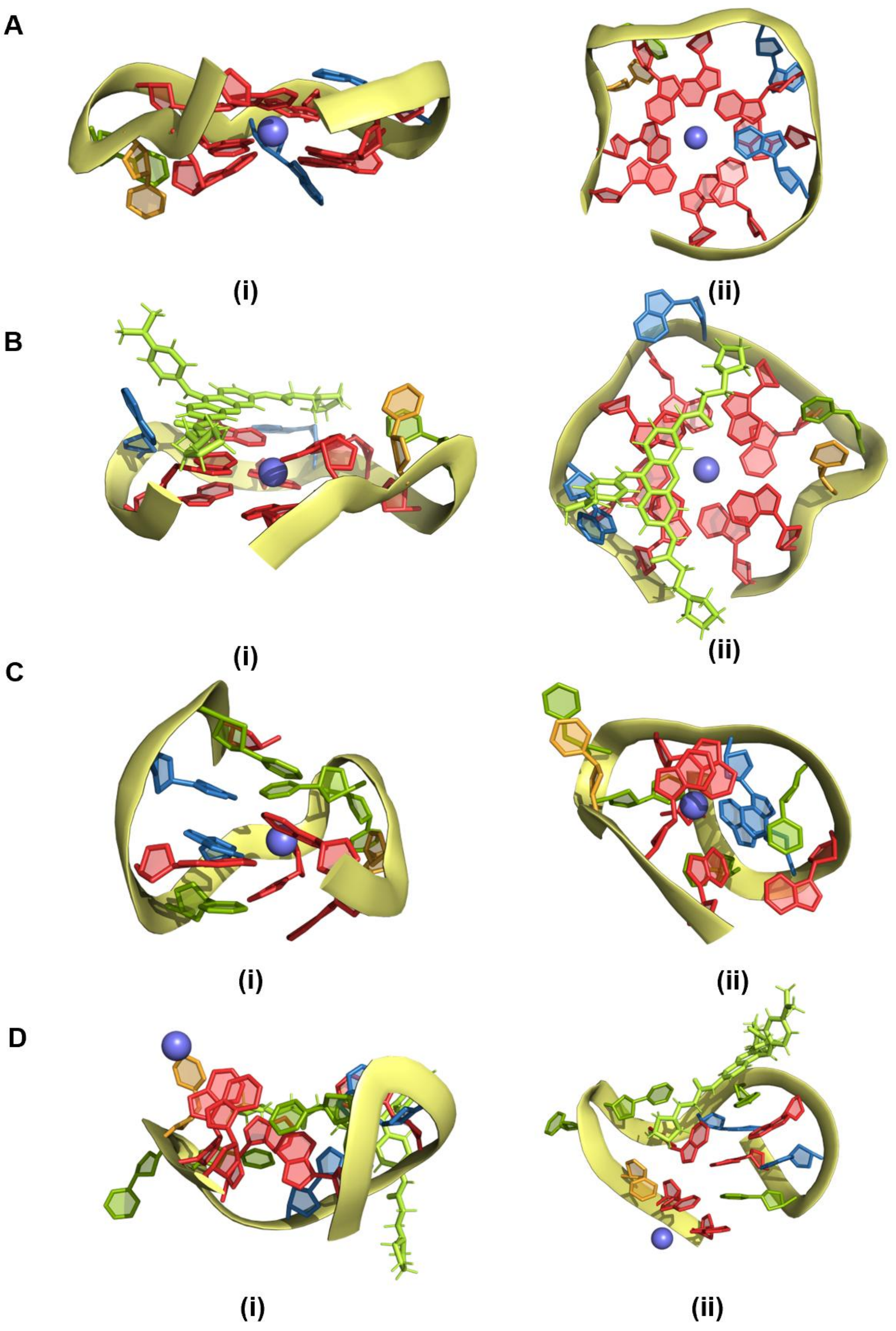

Figure S9. G4-3WT and G4-3 MT interaction analysis with BRACO-19

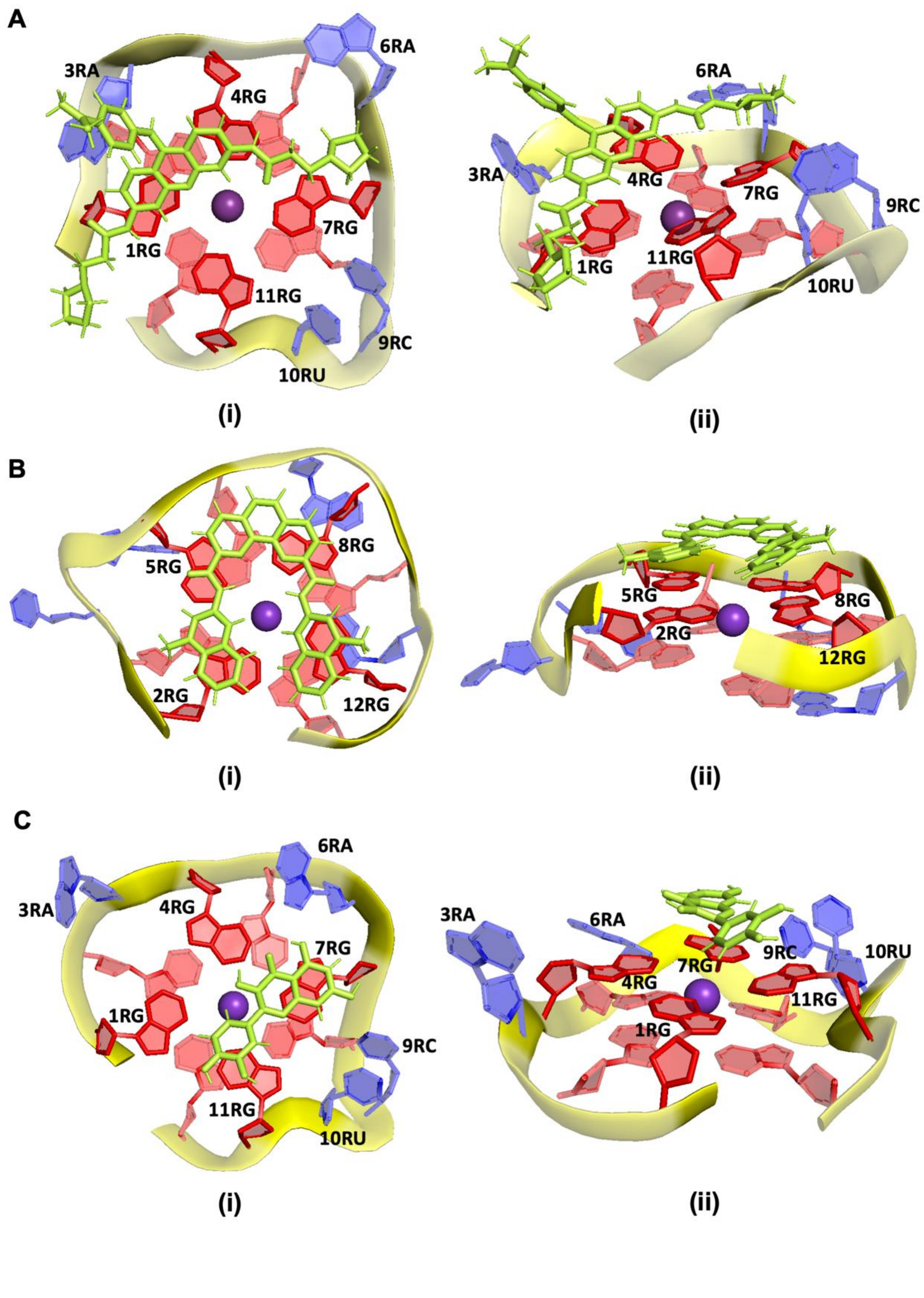

Figure S10. Comparing binding modes of Braco-19, PhenDC3 and Quercetin to G4-3

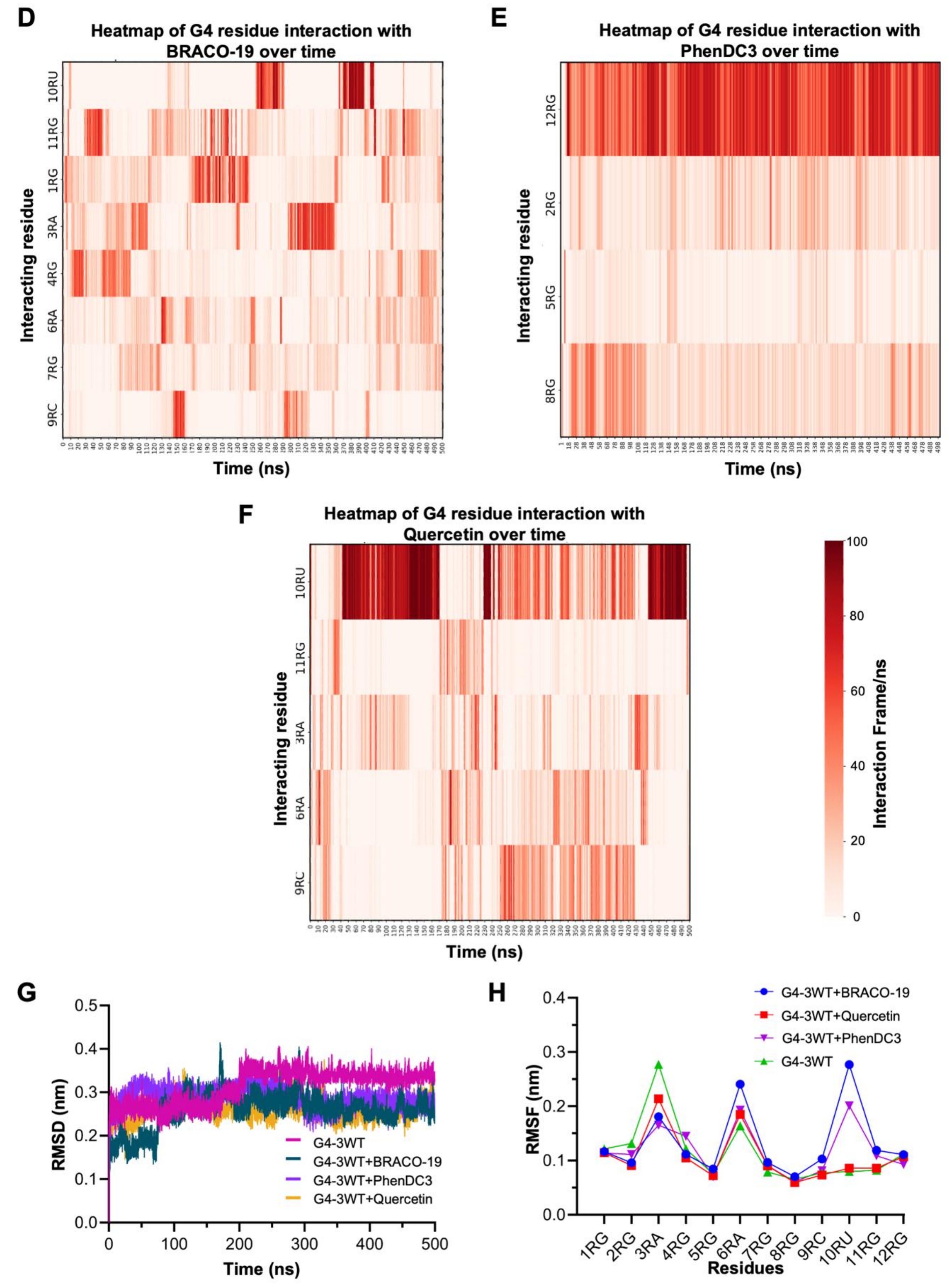

Figure S10. Comparing binding modes of Braco-19, PhenDC3 and Quercetin to G4-3

Sl. No.	Start	End	Score	Strand	Sequence (5'-3')
1	976	989	29	+	GGGGTTTCAGGAGG
2	1234	1247	29	+	GGATGGGGAAATGG
3	2523	2537	27	-	CCAACCAGAATCCCC
4	4543	4556	29	-	CCTTCACCCCCACC
5	4759	4770	32	+	GGAGGAGGCTGG
6	5247	5259	30	-	CCAGACCCCAGCC
7	6480	6492	30	+	GGCAGGTGGAAGG
8	6570	6612	37	+	GGGAGGGATCTTTTTATTCTTGATGAG CGGAAGGGGCATAGGG
9	7816	7830	27	+	GGCAGAGGAGGCTGG
10	8956	8971	26	+	GGGGAATTCGGCAAGG
11	10345	10360	26	-	CCTGTGAGCCCCGTCC

Table S1. Position and sequence of eleven G4 sequences screened using pqsfinder

Sl. No.	Name	RNA Sequence (5'-3')
1	G4-1	GGGGUUUCAGGAGG
2	G4-2	GGAUGGGGAAAUGG
3	G4-3	GGAGGAGGCUGG
4	G4-4	GGCAGGUGGAAGG
5	G4-5	GGGAGGGAUCUUUUUAUUCUUGAUGAG CGGAAGGGGCAUAGGG
6	G4-6	GGCAGAGGAGGCUGG
7	G4-7	GGGGAAUUCGGCAAGG
8	NRAS (Positive Control)	GGGAGGGGCGGGUCUGGG

Table S2. Sequence of the seven RNA G-quadruplex and positive control sequences used for further study

	Flaviviruses	Reference IDs
**1**	Absettarov virus	KJ000002.1
**2**	Aedes flavivirus	KC181923.1
**3**	Alkhumra hemorrhagic fever virus	AF331718.1
**4**	Aroa virus	KF917535.1
**5**	Bagaza virus	KR108246.1
**6**	Banzi virus	DQ859056.1
**7**	Barkedji virus	KC496020.1
**8**	Bouboui virus	DQ859057.1
**9**	Bussuquara virus	NC_009026.2
**10**	Cacipacore virus	NC_026623.1
**11**	Chaoyang virus	NC_017086.1
**12**	Culex theileri flavivirus	HE574574.1
**13**	Culiseta flavivirus	KT599442.1
**14**	Donggang virus	JQ086551.1
**15**	Edge Hill virus	DQ859060.1
**16**	Entebbe bat virus	DQ837641.1
**17**	Gadgets Gully virus	DQ235145.1
**18**	Hanko virus	JQ268258.1
**19**	Iguape virus	AY632538.4
**20**	Ilheus virus	KC481679.1
**21**	Japanese encephalitis virus	L48961.1
**22**	Jugra virus	DQ859066.1
**23**	Kama virus	NC_023439.1
**24**	Kamiti River virus	NC_005064.1
**25**	Karshi virus	DQ462443.1
**26**	Kedougou virus	NC_012533.1
**27**	Kokobera virus	NC_009029.2
**28**	Koutango virus	OQ067500.1
**29**	Kyasanur Forest disease virus	AY323490.1
**30**	Lammi virus	FJ606789.2
**31**	Langat virus	NC_003690.1
**32**	Meaban virus	DQ235144.1
**33**	Modoc virus	AJ242984.1
**34**	Murray Valley encephalitis virus	NC_000943.1
**35**	Nakiwogo virus	GQ165809.1
**36**	Naranjal virus	KF917538.1
**37**	New Mapoon virus	KC788512.1
**38**	Nhumirim virus	NC_024017.1
**39**	Nienokoue virus	NC_024299.1
**40**	Nounane virus	EU159426.2
**41**	Ntaya virus	NC_018705.3
**42**	Omsk hemorrhagic fever virus	AY193805.1
**43**	Palm Creek virus	KC505248.1
**44**	Potiskum virus	DQ859067.1
**45**	Powassan virus	KU886216.1
**46**	Quang Binh virus	NC_012671.1
**47**	Rio bravo virus	NC_003675.1
**48**	Kadam virus from Uganda	NC_033724.1
**49**	Royal Farm virus	DQ235149.1
**50**	Saboya virus	DQ859062.1
**51**	Saumarez Reef virus	DQ235150.1
**52**	Sepik virus	NC_008719.1
**53**	Spondweni virus	NC_029055.1
**54**	Sokuluk virus	NC_026624.1
**55**	St. Louis encephalitis virus	NC_007580.2
**56**	Stratford virus	KF917540.1
**57**	Tamana bat virus	AF285080.1
**58**	Tick-borne encephalitis virus	NC_001672.1
**59**	Uganda S virus	DQ859065.1
**60**	Usutu virus	MT795154.1
**61**	Wesselsbron virus	NC_012735.1
**62**	West Nile virus	AY646354.1
**63**	Xishuangbanna aedes flavivirus	KU201526.1
**64**	Yaounde virus	EU082199.2
**65**	Yellow fever virus	NC_002031.1
**66**	Yokose virus	AB114858.1
**67**	Zika virus	NC_012532.1

Table S3. The panel of 67 flaviviruses used for G4 conservation analysis

Flaviviruses	Abbreviation	Reference IDs
Dengue virus serotype 1	DENV1	NC_001477.1
Dengue virus serotype 2	DENV2	NC_001474.2
Dengue virus serotype 3	DENV3	NC_001475.2
Dengue virus serotype 4	DENV4	NC_002640.1
Japanese encephalitis virus	JEV	L48961.1
West Nile virus	WNV	AY646354.1
Murray Valley encephalitis virus	MVEV	NC_000943.1
Zika virus	ZIKV	NC_012532.1
Yellow fever virus	YFV	NC_002031.1
Sepik virus	SEPV	NC_008719.1
Wesselsbron virus	WESSV	NC_012735.1
Tick-borne encephalitis virus	TBEV	NC_001672.1

Table S4. List of flaviviruses with their reference IDs used for G4 conservation analysis

Figure #	Virus	Titer (FFU/ml)
2	DENV2	$5.0 X 10^5$
3	DENV2	$5.0 X 10^5$
5	rDENV2-G4-3WT	$2.0 X 10^6$
	rDENV2-G4-3WT	$1.0 X 10^6$
6	DENV2	$2.0 X 10^7$ (Concentrated)

Table S5. Titer of DENV used in this study
